# PFKFB3 Inhibition Sensitizes DNA Crosslinking Chemotherapies by Suppressing Fanconi Anemia Repair

**DOI:** 10.3390/cancers13143604

**Published:** 2021-07-18

**Authors:** Anna Huguet Ninou, Jemina Lehto, Dimitrios Chioureas, Hannah Stigsdotter, Korbinian Schelzig, Emma Åkerlund, Greta Gudoityte, Ulrika Joneborg, Joseph Carlson, Jos Jonkers, Brinton Seashore-Ludlow, Nina Marie Susanne Gustafsson

**Affiliations:** 1 Science for Life Laboratory, Department of Oncology and Pathology, Karolinska Institutet, 171 21 Stockholm, Sweden; anna.huguet@ki.se (A.H.N.); jemina.lehto@ki.se (J.L.); dimitrios.chioureas@ki.se (D.C.); Hannah.Stigsdotter@scilifelab.se (H.S.); korbinian.schelzig@gmx.de (K.S.); emma.akerlund@ki.se (E.Å.); greta.gudoityte@ki.se (G.G.); brinton.seashore-ludlow@ki.se (B.S.-L.); 2 Kancera AB, Karolinska Science Park, 171 48 Solna, Sweden; 3 Department of Women’s and Children’s Health, Karolinska Institutet, 171 21 Stockholm, Sweden; ulrika.joneborg@ki.se; 4 Department of Oncology and Pathology, Karolinska Institutet, 171 76 Stockholm, Sweden; joseph.carlson@ki.se; 5 Department of Pathology and Laboratory Medicine, Keck School of Medicine, University of Southern California, Los Angeles, CA 90089, USA; 6 Oncode Institute and Division of Molecular Pathology, The Netherlands Cancer Institute, 1066CX Amsterdam, The Netherlands; j.jonkers@nki.nl

**Keywords:** PFKFB3, Fanconi anemia pathway, KAN0438757, DNA repair, FANCD2

## Abstract

**Simple Summary:**

DNA-damaging chemotherapeutics, such as platinum drugs, are cornerstones in cancer treatment. The efficacy of such treatment is intimately linked to the DNA repair capacity of the cancer cells, as DNA damage above a tolerable threshold culminates in cell death. Cancer cells often have deregulated DNA repair mechanisms, making them initially more sensitive to DNA-damaging chemotherapies. Unfortunately, over time, cancer cells often develop resistance to such treatments by rewiring their DNA damage response pathways. Here, we identify that targeting the recognized anti-cancer target 6-phosphofructo-2-kinase/fructose-2,6,-bisphophatase 3 (PFKFB3), commonly overexpressed in cancer, with the small molecule inhibitor KAN0438757, selectively sensitizes cancer cells to platinum drugs, including treatment-resistant cancer cells, while sparing normal cells. Mechanistically, PFKFB3 promotes tolerance to and the repair of platinum-induced DNA interstrand crosslinks (ICLs) through modulation of the Fanconi anemia (FA) DNA repair pathway. Thus targeting PFKFB3 opens up therapeutic possibilities to improve the efficacy of ICL-inducing cancer treatments.

**Abstract:**

Replicative repair of interstrand crosslinks (ICL) generated by platinum chemotherapeutics is orchestrated by the Fanconi anemia (FA) repair pathway to ensure resolution of stalled replication forks and the maintenance of genomic integrity. Here, we identify novel regulation of FA repair by the cancer-associated glycolytic enzyme PFKFB3 that has functional consequences for replication-associated ICL repair and cancer cell survival. Inhibition of PFKFB3 displays a cancer-specific synergy with platinum compounds in blocking cell viability and restores sensitivity in treatment-resistant models. Notably, the synergies are associated with DNA-damage-induced chromatin association of PFKFB3 upon cancer transformation, which further increases upon platinum resistance. FA pathway activation triggers the PFKFB3 assembly into nuclear foci in an ATR- and FANCM-dependent manner. Blocking PFKFB3 activity disrupts the assembly of key FA repair factors and consequently prevents fork restart. This results in an incapacity to replicate cells to progress through S-phase, an accumulation of DNA damage in replicating cells, and fork collapse. We further validate PFKFB3-dependent regulation of FA repair in ex vivo cultures from cancer patients. Collectively, targeting PFKFB3 opens up therapeutic possibilities to improve the efficacy of ICL-inducing cancer treatments.

## 1. Introduction

The cellular pathways that promote the repair of DNA lesions and replication fork integrity are essential for genome stability [1]. Consequently, DNA damage levels above a tolerable threshold culminate in cell death. This is exploited therapeutically as cancer cells often have deregulated DNA repair mechanisms, making them more susceptible to DNA-damaging chemotherapies. DNA-crosslinking chemotherapies, such as platinum-based drugs and mitomycin C (MMC), exert their toxicity by introducing DNA interstrand crosslinks (ICLs) which block strand separation and impede processes requiring DNA unwinding. ICLs that hinder replication fork progression are repaired through the Fanconi anemia (FA) pathway [2]. Mutations in any of the to-date identified 22 FA genes results in FA, a rare recessive genetic disorder characterized by bone marrow failure, chromosome instability, predisposition to cancer, and cellular hypersensitivity to ICL-inducing agents [2,3].

The structure of a stalled replication fork at an ICL is detected by the DNA helicase and translocase FANCM [4], which, in concert with its interacting partners FAAP24 and MHF proteins [5], drives the assembly of the FA core complex, consisting of eight additional FA proteins (FANCs) [2]. The recruitment of FANCM to stalled forks is dependent on its own translocase activity, interaction with FAAP24, and the Bloom (BLM) helicase complex, and is stimulated by the activity of Ataxia telangiectasia and Rad3-related protein (ATR), independent of its interaction with the FA core complex [6,7,8,9,10]. FANCM and BLM, as well as ATR and FANCM, form a positive feedback loop to promote replication fork stability, reinforcing the recruitment and activity of each other [6,9,10,11,12]. The FA core complex promotes ubiquitination of the FANCD2:FANCI heterodimer upon its association to DNA at the stalled fork [13], an event that can be visualized as FANCD2 nuclear foci, which is considered a hallmark of FA pathway activation [14,15,16,17]. The heterodimer coats the DNA to protect the stalled replication forks [16,18] and recruits nucleases to incise the ICL leading to the unhooking of one of the parental strands and the induction of a DNA double strand break (DSB) [19]. The DSB intermediates are repaired via homologous recombination (HR) and the remaining strand with the ICL undergoes translesion synthesis [2].

The enzyme 6-phosphofructo-2-kinase/fructose-2,6,-bisphophatase 3 (PFKFB3) is commonly overexpressed in cancer where it promotes angiogenesis, migration, and proliferation [20,21,22]. More recent studies have shown the involvement of cytoplasmic PFKFB3 in response to DNA-damaging cisplatin in cervical cancer cells [23] and UV damage in primary mouse embryonic fibroblast [24]. Although PFKFB3 is a bifunctional enzyme in glycolysis and catalyzes the synthesis and degradation of the allosteric modulator Fructose 2,6-bisphosphate [20], PFKFB3 has the capacity to drive cancer cell proliferation from its nuclear localization without modulation of glucose metabolism [20]. We previously reported that PFKFB3 relocates to DSBs upon ionizing radiation (IR), allowing for the assembly of key HR repair proteins into foci, required for functional DNA repair and cancer cell survival upon IR [25]. Consistent with a role in HR repair, PFKFB3 was recently reported to regulate HR repair of cisplatin-induced DSBs [26].

Here, we uncover a novel function of PFKFB3 in regulating FA repair, promoting tolerance to and repair of DNA crosslinks, distinct from its role in HR repair [25,26]. Upon FA pathway activation, ATR triggers the assembly of PFKFB3 into nuclear foci where PFKFB3 interacts with γH2AX and FANCD2 to enable the resolution of ICLs. Inhibition of PFKFB3 leads to the defective recruitment of FANCM, BLM, FANCD2, and FANCI repair proteins, which are essential for successful ICL repair during replication, recovery of DNA synthesis upon fork stalling, and cancer cell survival upon treatment with ICL-inducing agents. This modulatory mechanism of the FA response is associated with a PFKFB3 tumorigenic pattern of expression, as the synergistic anti-proliferative effect between PFKFB3 inhibition and crosslinking agents, carboplatin, and cisplatin, is specific to transformed cells.

## 2. Materials and Methods

### 2.1. Cell Culture

PA-1 (CRL-1572), Caov-3 (HTB-75), SW626 (HTB-78) and SK-OV-3 (HTB-77), HEK293T, U2OS and VH10 cell lines were acquired from the American Type Culture Collection (ATCC^®^, Manassas, VA, USA)). A2780 (93112519) and A2780 cis (93112517) cell lines were purchased from the European Collection of Authenticated Cell Cultures (ECACC, Sigma-Aldrich, St. Louis, MO, USA). The PEO1 and PEO1-C4.2 cells (cell line identity confirmed by Sanger sequencing) were a gift from K. Sanjiv (Karolinska Institute) and BJ-hTERT, BJ-SV40, and BJ-RasV12 cells [27] were a kind gift from W. Hahn (Dana-Farber Cancer Institute). Caov-3 cells were maintained in DMEM GlutaMAX (#31966-021, Thermo Fisher Scientific, Waltham, MA, USA), SW626 in Leibovitz L-15 (#11415-049. Thermo Fisher Scientific), and SK-OV-3 in McCoy 5A (#36600-021, Thermo Fisher Scientific) media. PA-1, BJ-hTERT, BJ-SV40, BJ-RasV12, VH10, and U2OS cells were cultured in DMEM with GlutaMAX (#31966-021, Thermo Fisher Scientific). PEO1, and PEO1-C4.2, A2780, and A2780Cis were grown in RPMI 1640 with GlutaMAX (#61870, Thermo Fisher Scientific) and HEK293T in Iscove’s Modified Dulbecco’s Medium (IMDM; #12440, Thermo Fisher Scientific). Ten percent fetal bovine serum (FBS; #10500, Thermo Fisher Scientific) and 5000 U/mL penicillin–5000 µg/mL streptomycin antibiotics (#15070, Thermo Fisher Scientific) were used as a supplement in all media, except for BJ-hTERT, BJ-SV40, BJ-RasV12 cells (5% FBS). Cells were grown at 37 °C in 5% CO_2_ in humidified incubators.

### 2.2. Tumor Samples from High-Grade Ovarian Cancer Patients

This study was conducted in compliance with the ethical requirements from Helsinki II Declaration and approved by the Stockholm regional division of the Swedish Ethical Review Agency (Etikprövningsmyndigheten) (ethical permits nr.2016/1197–31/1, nr.2018/2642-32 and nr.2018/118-32). Written informed consent was obtained from all participants before being included in the study. Omental tumor tissues were collected at Karolinska University Hospital and processed immediately upon receipt. Samples were washed in UltraSaline A solution (Lonza, #12-747F), cut to 2–4 mm pieces, and homogenized by employing gentleMACS™ (Miltenyi Biotec) tissue dissociator following manufacturer recommendations. Cell suspensions were filtered through a 70-µm strainer and centrifuged at 500 G for 5 min at RT. Remaining red blood cells were removed using ammonium-chloride-potassium lysis buffer following a wash-out with RPMI media supplemented with 10% FBS. Cell suspensions were plated in F-medium (Ham’s F-12 Nutrient Mixture:DMEM 3:1(*v/v*), 5% FBS, 0.4 µg/mL hydrocortisone, 5 µg/mL insulin, 8.4 ng/mL cholera toxin, 10 ng/mL EGF, 24 µg/mL adenine) supplemented with 10 µM ROCK inhibitor (Y-27632, Enzo Life Sciences, Lausen, Switzerland), and 7.5 µg/mL transferrin (#T3309, Sigma-Aldrich), and were grown at 37 °C in 5% CO_2_ in humidified incubators. After recovery, patient-derived cells were cultured in DMEM GlutaMAX (#31966-021, Thermo Fisher Scientific), and supplemented with 5% FBS (#10500, Thermo Fisher Scientific) and 5000 U/mL penicillin–5000 µg/mL streptomycin antibiotics (#15070, Thermo Fisher Scientific).

### 2.3. Plasmid and siRNA Transfections

Cells were seeded and transfected the following day or reverse transfected with oligonucleotides (10 nM), targeting human FANCM: siFANCM#1 (Thermo Fisher, #s33621) and siFANCM#2 (Termo Fisher, #s33619) previously validated in Ref [28], PFKFB3: siPFKFB3#1 (Ambion, 4390824, ID:s10357) and siPFKFB3#2 (Ambion, 4390824, ID:s10358) previously validated in Ref [25], or non-targeting control (Qiagen #1027281) using INTERFERin^®^ (Polyplus transfections^®^, #409-10) according to the manufacturer’s protocol. Empty vector and FLAG-PFKFB3 plasmids [29] were transfected using JetPEI^®^ (Polyplus transfections^®^, #101-10N) according to the manufacturer’s protocol.

### 2.4. Establishment of shRNA Cell Lines

Cloning of hairpin constructs and lentiviral particle production using individual hairpin constructs targeting PFKFB3 (shPFKFB3-sh#1:TRCN0000007338, sh#2: TRCN0000007340, sh#3:TRCN0000314690, and sh#4:TRCN0000314746; and shScramble control) was performed as previously described [30] as well as the establishment of shRNA cell lines

### 2.5. Quantitative Real-Time PCR (qRT-PCR) Analysis

Collection of cells, RNA extraction, cDNA synthesis, and qPCR reactions were carried out, as previously described [30]. The following primers were used: PFKFB3 (Forward: CAGTTGTGGCCTCCAATATC, Reverse: GGCTTCATAGCAACTGATCC) and β-actin (forward: CCTGGCACCCAGCACAAT, Reverse: GGGCCGGACTCGTCATACT) primers. Relative mRNA levels were calculated by employing the 2-ΔΔCT method [31] in relation to β-actin.

### 2.6. Small-Molecule Inhibitors and Cytostatic Compounds

PFKFB3 inhibitor KAN0438757 [25] (Kancera AB), Aphidicolin (Sigma-Aldrich, #A0781), ATR inhibitor VE-82154 [32] (Axon MedChem, #Axon1893), and RPA inhibitor TDRL505 [33] (Sigma-Aldrich, #5.30535) were dissolved in dimethyl sulfoxide (DMSO; Sigma-Aldrich, #41369). Cisplatin (Sigma-Aldrich, #P4394) and carboplatin (SelleckChem, #S1215) were dissolved in 0.9% NaCl or PBS. 2-DeoxyGlucose (Sigma-Aldrich, #D8375) and Sodium Oxamate (Sigma-Aldrich, #O2751) were dissolved in cell media. Mitomycin C (MMC; Santa Cruz Biotechnology, #sc-3514B) was dissolved in H_2_O.

### 2.7. Cell Growth Kinetics

shPFKFB3 and shScramble A2780 cells were seeded at concentration 2 × 10^3^ cells per well in 96-well plates. The next day, cells were treated as indicated and subjected to real-time bright field imaging (Incucyte^®^ S3; Essen Bioscience, Inc., Ann Arbor, MI, USA) for 168 h, as previously described [30].

### 2.8. End-Point Cell Viability Assays

Platinum compounds were dispensed manually and KAN0438757 was dispensed using Tecan D300 (Tecan, Hewlett-Packard, CA, USA) on 96-well plates with normalization for DMSO content. Cells (2 × 10^4^ to 4.5 × 10^4^ per well) were plated and incubated for 72 h. For the assessment of the effect of glucose levels for cell viability upon carboplatin treatment, glucose-free DMEM (Thermo Fischer, #A1443001) supplemented with 10% FBS and 1% P/S was used. Viability was measured using 100 µg/mL resazurin sodium salt (Sigma-Aldrich, #R7017), followed by fluorescence 544 nm / 590 nm measurement using a Hidex Sense (Hidex Oy) microplate reader. Viability percentages were calculated in relation to DMSO control set as 100% viability value. Dose-response curves were generated using GraphPad Prism software (version 8.1.1) and EC_50_ values were obtained by non-linear sigmoidal curve fitting. Drug synergy experiments were performed, as previously described [30].

### 2.9. Clonogenic Assays

Clonogenic assays were performed, as previously described [30], with the following modification. Cells were treated with compounds as indicated, compounds were washed out with fresh media after 24 h co-treatment, and cells kept in culture for 10 days prior fixation.

### 2.10. Extracellular Flux Analysis

The cellular oxygen consumption rate (OCR) and extracellular acidification rate (ECAR) were measured with XF96 extracellular flux analyzer (Seahorse Bioscience). Cells were seeded in triplicates on XFe96 Cell culture Microplates (Seahorse, Bioscience #100085-004) in unbuffered DMEM containing 1 mM glucose, without pyruvate, glutamate, and FBS. Prior to the experiment, cells were incubated in a CO_2_-free incubator at 37 °C for 2 h to allow for temperature and pH equilibration. The experiment consisted of sequential mix (3 min) and measurement (3 min) cycles, providing data every 6 min for determination of OCR and ECAR. Reagents or media were sequentially injected, as indicated through the assay.

### 2.11. Flow Cytometry for Cell Cycle Progression of Replicating Cells

Cell cycle progression of replicating cells was assayed using a modified method, as previously described [30], with the following modifications; cells were seeded and the following day they were synchronized with 24 h treatment of 6 µM aphidicolin, released into media containing 10 µM 5-Ethynyl-2’-deoxyuridine (EdU) (Sigma-Aldrich, #1T511285) for 45 min, followed by treatment with 10 µM cisplatin alone or in combination with 5 µM PFKB3i for 6 h or 16 h. Post blocking, cells were incubated with primary antibody γH2AX-Ser139 (Millipore, #05-636, 1:500) overnight at 4 °C. Cells were washed twice with 1% BSA/PBS solution and incubated with secondary antibody goat anti-mouse Alexa 405 (1:50, #A31553, Invitrogen, Carlsbad, CA, USA) for 30 min at 37 °C followed by Click-iT reaction.

### 2.12. Immunoblotting and Whole Cell Protein Extraction

Cells were seeded on 6-well plates, left to attach for 24 h, and then treated as indicated. Cells were lysed with NP-40 lysis buffer of 50 mM Tris-HCl pH 8.0 (Sigma-Aldrich, #T1503), 150 mM NaCl (EMD Millipore, #31434-M), 1% NP-40 (BioVision, #2111), and supplemented with protease (Roche, #04693159001) and phosphatase inhibitors (Thermo-Fisher Scientific, #78426). Protein lysates were quantified using the Pierce BCA Protein Assay Kit (ThermoFisher Scientific, #23225) and the normalized total protein amount. For immunoblotting, samples were processed, as previously described [30]; the antibodies used are listed in Table S1.

### 2.13. Cell Fractionation of Whole Cell Soluble and Chromatin Bound Proteins

One million cells were seeded on 100 mm plates and the following day were treated as indicated. Cells were trypsinized and the equal number of cells were processed, as previously described [30].

### 2.14. Immunoprecipitations

Five hundred thousand U2OS cells were seeded in 100 mm plates and were transfected with 1 µg FLAG-PFKFB3 plasmid or empty vector or left untransfected the following day. Twenty-four hours later, cells were treated as indicated. Cells were subject to chromatin fractionation (see section above) or lysed into whole cell lysates for protein interactions, as previously described [34]. Immunoprecipiations were performed with Dynabeads™ Protein G according to the manufacturer’s recommendation (Fisher, # 10765583). Antibodies were used at a concentration of 2 μg/sample and included mouse anti-FLAG (Sigma-Aldrich, #F3165), rabbit anti-PFKFB3 (Proteintech #13763-1-AP), rabbit anti-BLM (Bethyl Laboratories #A300-110A-M), or species-matched IgG controls.

### 2.15. Fiber Assay

Recovery of fork progression after replication stalling was assayed using a modified method, as previously described [25]. Briefly, cells were incubated with 25 µM CIdU (Sigma-Aldrich, #C6891) for 20 min, followed by the addition of 1 mM or 3 mM mitomycin C alone or in combination with 10 µM PFKFB3 inhibitor for 1 h. Next, cells were washed and incubated with 250 μM IdU (Sigma, #I7125) for 40 min. Following pulse labelling, cells were processed, as previously described [30] and fluorescence images were acquired with Zeiss LSM 780 microscope using a 40x oil immersion objective and analyzed using Fiji [35,36]. A minimum of 100 CIdU/IdU-labeled restarted replication forks were measured for every condition. Restarted, newly fired, and stalled forks were counted manually through each dataset. Antibodies used are listed in Table S1.

### 2.16. Immunofluorescence Microscopy

Forty thousand to fifty thousand cells were seeded in 12-well plates on 18 mm sterilized coverslips (VWR #631-1580) and left to attach for 24 h. Cells were either treated as indicated to address effects on the unsynchronized cell population, or synchronized using 6 µM aphidicolin for 24 h. Cells were washed with PBS and processed, as previously described [30]. Images were acquired with a Zeiss LSM 780 microscope using a 40× oil immersion lens and processed in CellProfiler software (www.cellprofiler.org, last accessed on 12 July 2021) [37] for analysis. For confocal experiment with EdU labelling, cells were pulsed with 10 μM EdU (Sigma-Aldrich, #1T511285) for 30 min before fixation and Click-iT reaction was performed, as described above. Antibodies used are listed in Table S1.

### 2.17. Statistics

Statistical analyses were carried out using GraphPad Prism software (version 8.1.1) by Student’s *t*-test or two-tailed ANOVA. Multiple comparisons were performed with ordinary one-way ANOVA followed with Holm-Sidak’s correction or Dunnett test correction. Data are presented as means ± standard deviation (SD) or standard error of the mean (SEM) with a 95% confidence interval. * *p* < 0.05, ** *p* < 0.01, *** *p* < 0.001.

## 3. Results

### 3.1. Targeting PFKFB3 Enzymatic Activity Results in a Non-Reversible Cancer-Specific Synergy with Platinum Drugs

**Figure 1:**
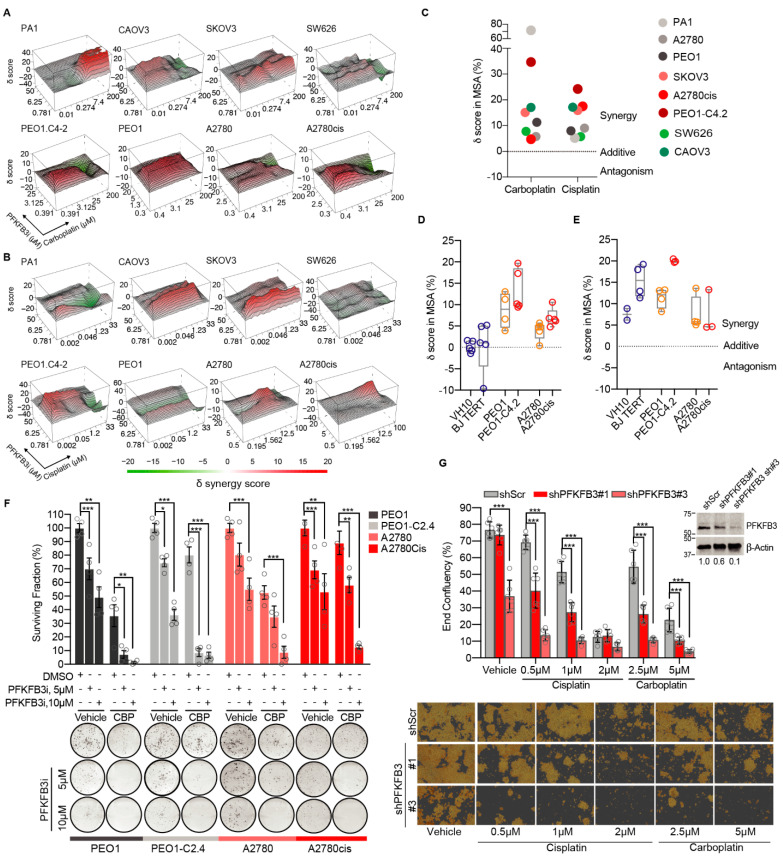
Targeting PFKFB3 enzymatic activity resulted in a non-reversible cancer-specific synergy with platinum drugs. (**A**–**B**) Three-dimensional heatmaps of the synergy surface of dose-response viability matrixes upon carboplatin (**A**) or cisplatin (**B**) in combination with PFKFB3 inhibitor (PFKFB3i) for 72 h. Representative graphs from initial screening across eight different cancer cell lines. Synergy scores were calculated by the zero interaction potency model and images were generated by Synergy Finder (version 2.0). (**C**) Scatter dot plot of delta scores in the most synergistic area (MSA) from each synergy matrix of (**A**) and (**B**). (**D**) Delta scores in the MSA upon carboplatin treatment in combination with PFKFB3i in non-transformed cell lines (depicted in blue), and platinum-sensitive (yellow) and resistant (red) epithelial ovarian cancer cell lines. Viability was evaluated at 72 h of drug treatments and synergy calculated by the zero interaction potency model. Data are displayed as Min to Max box plot, *n* ≥ 4. (**E**) Box plot of delta scores in the MSA upon 72 h treatment with carboplatin and 2-DeoxyGlucose (2-DG) in non-transformed cell lines (blue), and two pairs of isogenic cancer cell lines sensitive (yellow) and resistant (red) to platinum. Viability was calculated by the zero interaction potency model. Data are displayed as Min to Max box plot *n* ≥ 4. (**F**) Clonogenic survival of indicated cells lines treated as indicated for 24 h followed by 24 h co-treatment with 2.5 µM carboplatin (CBP) and then drug washout. After 10 days, colonies were stained and manually counted. Top panel, bars represent quantification of colony survival normalized to vehicle-treated cells (100%) per each cell line. Bottom panel, representative images of colony formation assays. Data are displayed as means ± S.D, *n* = 4, from 2 experiments. * *p* < 0.05, ** *p* < 0.01, *** *p* < 0.001; One-way ANOVA analysis, Sidak’s test for multiple comparisons. (**G**) Cell proliferation assay of shScramble, shPFKFB3#1 and shPFKFB3#3 A2780 cells routinely cultured with cisplatin (2 µM). Top panel shows end-point confluency determined by the occupied area (% confluence) by indicated cell lines treated for 7 days with cisplatin or carboplatin, or left untreated. Data represented as means ± SD of triplicates, each replicate determined by mean of *n* = 4 images; *** *p* < 0.001; One-Way ANOVA analysis, Holm-Sidak’s test for multiple comparisons. On the top-right, immunoblot of PFKFB3 expression levels in A2780 cells expressing indicated shRNAs targeting PFKFB3. β-Actin was used as a loading control. The values illustrate densitometric quantifications of PFKFB3 protein levels normalized to β-Actin and then relative to the value obtained for the shScr sample. Images of the uncropped western blots can be found in Figure S14. The bottom panel displays representative images of fields used to calculate end-point confluency, images acquired with 10× microscope magnification from Incucyte® SX5 Live-Cell Analysis System (Essen Bioscience, Inc., Ann Arbor, MI, USA).

To determine the contribution of PFKFB3 activity in the response to platinum, we performed drug combination screenings in a dose-response matrix across a panel of cancer cell lines using the PFKFB3 inhibitor KAN0438757 [25,38], hereafter referred to as PFKFB3i, given its inhibition of PFKFB3 enzymatic activity, proven target engagement, and isoenzyme selectivity [25]. Strong synergy scores between PFKFB3i and platinum-based drugs were observed in all cancer cell lines tested (Figure 1A–C). Given the clinical challenge of acquired platinum resistance and dose-limiting toxicity towards non-cancerous cells [39], PFKFB3i and carboplatin combination treatments were evaluated in non-transformed cell lines (BJTERT, VH10) in relation to two pairs of isogenic cancer cell line models of acquired platinum resistance: the platinum-sensitive PEO1 and A2780, and the resistant PEO1.C4-2 and A2780cis. The synergy scores between PFKFB3i and carboplatin were further increased in platinum-resistant cell lines in comparison to the sensitive ones, whereas the combination treatment did not result in any synergistic delta scores in the non-transformed cell lines (Figure 1D and Figure S1a). In contrast to PFKFB3i, combining the competitive glycolytic inhibitor 2-Deoxy-D-Glucose (2-DG) with carboplatin resulted in similar synergy scores in both cancer and non-transformed cells (Figure 1E). Furthermore, the interactions between PFKFB3i and carboplatin were non-reversible upon drug washout and restored sensitivity to carboplatin in the resistant cell models (Figure 1F). In line with this data, shPFKFB3 A2780 cells with acquired platinum resistance became re-sensitized to cisplatin and carboplatin treatments (Figure 1G, Figures S1b and S14). Taken together, inhibition of PFKFB3 enzymatic activity resulted in a non-reversible cancer-specific synergy with platinum drugs in both treatment-sensitive and resistant cell models in contrast to general inhibition of glycolysis which shows toxicity towards non-transformed cells.

### 3.2. Platinum Drugs Do Not Induce a Preference for Glycolysis

**Figure 2:**
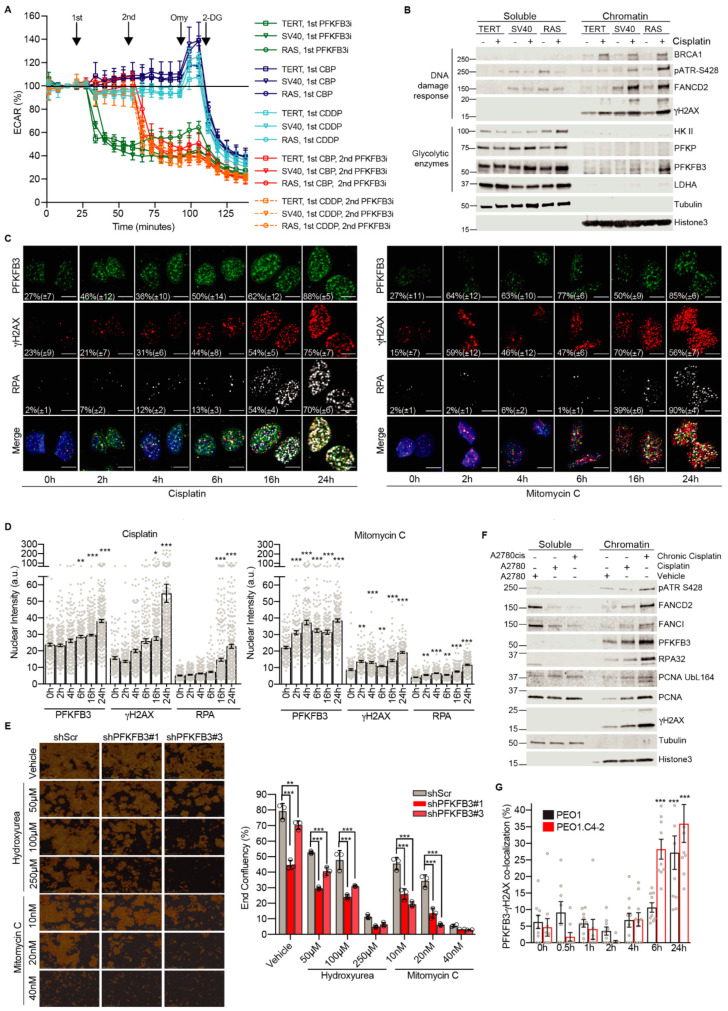
Cancer-specific PFKFB3 nuclear localization in response to FA pathway activation. (**A**) Extracellular acidification rate (ECAR) in BJ fibroblast transformation series; TERT (non-malignant), SV40 and RAS (malignant) cell lines. Cells were treated with either PFKFB3i (10 µM), carboplatin (CBP, 100 µM), or cisplatin (CDDP, 100 µM) at indicated time points, all samples were treated with 2 µM Oligomycin (Omy) to obtain maximal ECAR capacity and subsequently with 20 mM 2-deoxy glucose (2-DG) for a complete block of glycolysis. Data displayed as mean values at each time point ± S.D, *n* = 5. (**B**) Isolation of whole cell soluble and chromatin-bound proteins by fractionation and immunoblotting of BJ hTERT, SV40, and RAS cells treated with vehicle or 2.5 µM cisplatin for 24 h. Tubulin was used as a control for the soluble fraction and Histone 3 as a control for the chromatin-associated fraction. Representative blot of *n* > 3 experiments. Images of the uncropped western blots can be found in Figure S8. (**C**) Representative images of PFKFB3, γH2AX, and RPA32 nuclear recruitment kinetics upon treatment with cisplatin (10 µM) or Mitomycin C (360 nM) in U2OS cells. Cells were fixed at indicated time points post-treatment and immunostained. Data are represented as percentage of foci-positive cells ± S.D, representative of *n* = 2 independent experiments per treatment, *n* > 100 cells per condition. Quantification of the percentage of foci-positive cells was determined using CellProfiler by counting cells containing treatment-induced foci above the average of untreated (0 h) sample, scale bar of 10 µm. (**D**) Image-based quantification of PFKFB3, γH2AX, and RPA32 nuclear staining intensity in (**C**). Quantification was performed with CellProfiler. Single cell data displayed as individual points with bar graphs showing mean ± S.E.M. Representative of *n* = 2 experiments, *n* > 100 cells per time-point, *n* > 100 cells/treatment. * *p* < 0.05, ** *p* < 0.01, *** *p* < 0.001; ordinary one-way ANOVA analysis, Dunnett’s test for multiple comparisons. (**E**) Cell proliferation of A2780 cells expressing shScramble (SCR), shPFKFB3#1 or shPFKFB3#3 and treated as indicated for 7 days. On the left, representative images of fields used to calculate end-point confluency, images acquired with 10× microscope magnification from Incucyte^®^ SX5 Live-Cell Analysis System. To the right, the measurement of end-point confluency after treatment exposure. Data represent mean ± SD of triplicates, each replicate determined by mean of *n* = 4 images; ** *p* < 0.01, *** *p* < 0.001; one-way ANOVA analysis, Sidak’s test for multiple comparisons. (**F**) A2780 cell left untreated or treated with 2.5 µM cisplatin for 24 h and A2780cis cultured in 2.5 µM cisplatin were subjected to cell fractionations for isolation of whole cell soluble and chromatin-bound proteins followed by immunoblotting. Representative blot of *n* > 3 experiments. Images of the uncropped western blots can be found in Figure S10. (**G**) Confocal analysis of PFKFB3 recruitment kinetics at DNA damage sites upon carboplatin treatment in PEO1 and PEO1.C4-2 isogenic cell lines. Each cell line was treated with corresponding carboplatin EC_50_ concentrations: 12.3 µM for PEO1 and 25 µM for PEO1.C4-2 cells. Quantification of the percentage of cells with PFKFB3 and γH2AX co-localizing nuclear foci was performed employing CellProfiler. Data displayed as means ± S.E.M, representative of *n* = 2 experiments, *n* > 100 cells per time-point, *n* > 100 cells/treatment. *** *p* <0.001; ordinary one-way ANOVA, Dunnett’s test for multiple comparisons.

We next assessed whether the cancer-specific synergies between platinum drugs and PFKFB3i were due to PFKFB3i-mediated suppression of potential platinum-induced changes in glycolytic metabolism during cancer transformation. To this end, we employed the BJ fibroblasts transformation series immortalized by the expression of telomerase (TERT), transformed with the SV40 large-T antigen, and finally transduced with oncogene H-Ras (RASG12V) to become metastatic [27]. Even at the highest dose tolerated by the cells, cisplatin and carboplatin treatments did not result in significant changes in glycolysis as assessed by the extraCellular acidification rate (ECAR) between BJTERT, SV40, and RAS cells (Figure 2A). Furthermore, the three cell lines were equally affected by the addition of PFKFB3i which decreased ECAR to baseline levels to a similar extent as 2-DG treatment, indicating a block in the glycolysis upon PFKFB3i as expected. Similar results were obtained in the A2780 cell line (Figure S2a–c). In line with this data, alterations of glucose levels in the cell media did not affect sensitivity to carboplatin in viability experiments (Figure S2d). Collectively, PFKFB3i and platinum treatments do not induce differential responses in glycolysis across cancer transformation, thus the cancer-specific synergies between PFKFB3 inhibition and platinum drugs in viability assays are not due to differences in metabolic responses measured.

### 3.3. Cancer-Specific PFKFB3 Chromatin Localization in Response to FA Pathway Activation Modulates Platinum-Tolerance of Resistant Cells

Considering the synergy between PBKFB3i and DNA-damaging platinum drugs (Figure 1A–C) and the previously described relocation of PFKFB3 into nuclear foci at DNA damage sites upon induction of DNA DSBs [25], we next determined PFKFB3 intracellular localization upon cisplatin treatment during cancer transformation. Surprisingly, cisplatin triggered an accumulation of PFKFB3 in the chromatin-bound fraction in the transformed SV40 and RAS cells with an enhanced chromatin association in the RAS cells (Figure 2B and Figure S8). In contrast, PFKFB3 did not associate to the chromatin in the non-transformed BJTERT cells. PFKFB3 chromatin localization was validated using FLAG-tagged PFKFB3 as well as upon PFKFB3 siRNA, which abolished the PFKFB3 band in the chromatin fraction (Figures S3a,b and S9a–d). It is worth mentioning that glycolytic enzymes upstream of PFKFB3, hexokinase II (HKII) and phosphofructokinase (PFKP), or downstream, lactate dehydrogenase (LDHA), did not show any association to the chromatin (Figure 2B) but increased in the whole cell soluble fraction of the transformed cells upon cisplatin. The cisplatin-triggered chromatin enrichment of PFKFB3 in SV40 and RAS cells correlated with the recruitment of DNA damage response proteins, including breast cancer type 1 susceptibility protein (BRCA1), phosphorylated ATR, FANCD2 as well as the DNA damage marker phosphorylated H2A histone family member X at serine 139 (γH2AX).

We next assessed PFKFB3 foci formation in comparison to DNA damage markers by employing the U2OS cell line, a well-established cell line to study DNA repair with similar a EC_50_ value for cisplatin as A2780Cis cells (Figure S4a), since A2780Cis cells displayed poor attachment to microscope slides. Notably, an increased recruitment of PFKFB3 into nuclear foci was observed already after 2 h of cisplatin in comparison to γH2AX which appeared at 4 h followed by a slow increase in RPA up to 16 h. Between 16 h to 24 h, both γH2AX, PFKFB3, and RPA nuclear levels drastically increased, most likely due to DSB induction at these timepoints (Figure 2C,D). Given the pivotal role of the FA repair pathway in resolving cisplatin-induced DNA lesions, we extended our observations of PFKFB3 recruitment to other FA pathway-activating drugs. Similar to cisplatin, MMC increased PFKFB3 foci by 37% already at 2 h, while RPA foci significantly increased at 16 and 24 h, again indicative of DSB formation at this timepoint (Figure 2C,D). PFKFB3 recruitment upon MMC treatment was dose-dependent and also achieved with low dose hydroxyurea treatment known to activate FA repair [40] (Figure S4b,c). In support of a potential function of PFKFB3 in FA repair, knockdown of PFKFB3 sensitized cells to low dose hydroxyurea and MMC in proliferation assays (Figure 2E). In line with FA pathway activation being associated with platinum resistance [41,42,43,44], PFKFB3 translocation to chromatin upon cisplatin was further enhanced in the resistant A2780Cis cells (Figure 2F and Figure S10) which correlated with increased activation of the FA pathway as assessed by the recruitment of phosphorylated ATR, FANCD2, FANCI, RPA32, the proliferating cell nuclear antigen (PCNA), and its monoubiquitination at Lysine 164, which were both required for translesion synthesis, and γH2AX. In addition, platinum-resistant PEO1.C4-2 cells displayed faster recruitment kinetics of PFKFB3 into foci upon carboplatin treatment in comparison to PEO1 (Figure 2G and Figure S4d). Altogether, PFKFB3 is uniquely loaded to chromatin upon cisplatin treatment during cancer transformation with increased association and faster kinetics into foci upon platinum resistance, which correlates with a potential role in FA repair.

### 3.4. PFKFB3 Is Recruited into Foci upon Initiation of Replicative S-Phase FA Repair by FANCM and ATR

**Figure 3:**
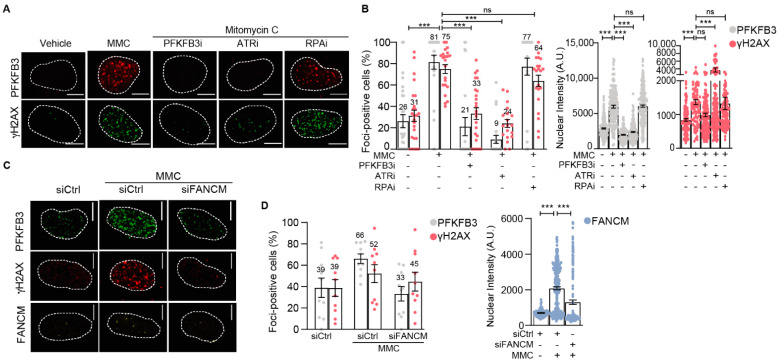
PFKFB3 requires ATR kinase activity for its nuclear recruitment to repair ICL-induced damage in replicating cells. (**A**) Representative confocal images of PFKFB3 and γH2AX foci in U2OS cells synchronized at the G1/S boundary and released for 3 h in Mitomycin C (MMC, 360 nM) with or without PFKFB3i (10 µM), ATRi (2.5 µM), or RPAi (50 µM), *n* ≥ 2 independent experiments. Dotted lines indicate the nuclear border. Scale bar, 10 μm. (**B**) Scatter dot plot with bar charts of percentage PFKFB3 and γH2AX foci positive cells upon image-based analysis of (**A**). Left panel shows the percentage of cells containing number of nuclear foci above the average in vehicle, data displayed as means ± S.E.M, one data point represents foci-positive percentage per image. Right graphs show nuclear intensity of PFKFB3 and γH2AX stainings, data displayed as scatter dot plot with means ± S.E.M, one data point represents one cell. *n* ≥ 2 independent experiments, *n* > 100 cells per condition. *** *p* < 0.001; ordinary one-way ANOVA, Sidak’s test for multiple comparisons. (**C**) Representative confocal images of FANCM, PFKFB3, and γH2AX foci in U2OS cells treated with siFANCM or siControl for 24 h, followed by cell cycle synchronization at the G1/S boundary and released into S-phase for 3 h in Mitomycin C (MMC, 360 nM) or left untreated, *n* > 2 independent experiments. Scale bar, 10 μm. (**D**) Quantification of FANCM, PFKFB3, and γH2AX from (**C**) using CellProfiler. Left panel depicts the percentage of cells containing number of nuclear foci above the average in vehicle, data displayed as means ± S.E.M, one data point represents foci-positive percentage per picture. Right graph shows nuclear integrated intensity of FANCM. Data displayed as scatter dot plot with means ± S.E.M, one data point represents one cell. *n* > 2 independent experiments, *n* > 100 cells per condition. *** *p* < 0.001; ordinary one-way ANOVA, Sidak’s test for multiple comparisons.

We next assessed the mechanism by which FA pathway activation triggers assembly of PFKFB3 into foci. Cells were synchronized at the G1/S boundary by aphidicolin treatment followed by the release into vehicle or cisplatin to enrich the S cell population at the time point when ICL resolution occurs exclusively by elicitation of the FA pathway (Figures S5a,b and S11a,b). We observed a strong increase in the percentage of cells with PFKFB3 and γH2AX foci upon MMC exposure which was dependent on ATR activity and FANCM presence (Figure 3 and Figure S5c,d), consistent with the upstream role of ATR and FANCM in the FA pathway [9]. Moreover, the formation of both PFKFB3 and γH2AX into foci upon MMC was dependent on PFKFB3 activity (Figure 3A,B and Figure S5c). The dependence on ATR and PFKFB3 activity for PFKFB3 foci assembly was also confirmed in unsynchronized cells treated for 3 h with MMC (Figure S5d). Given that FANCM regulates the activation of checkpoint signaling upon ICL by recruiting RPA into foci for activation of ATR, independent from the role of RPA in DSB resection [10,11,12,45], we assessed foci recruitment upon RPA inhibition. The MMC-dependent recruitment of PFKFB3 and γH2AX was not dependent on RPA activity (Figure 3A,B and Figure S5c). Taken together, PFKFB3 is recruited into foci upon initiation of replicative S-phase FA repair by FANCM, ATR and its own activity.

### 3.5. PFKFB3 Kinase Activity Promotes Assembly of FA Factors into Foci at the Chromatin

**Figure 4:**
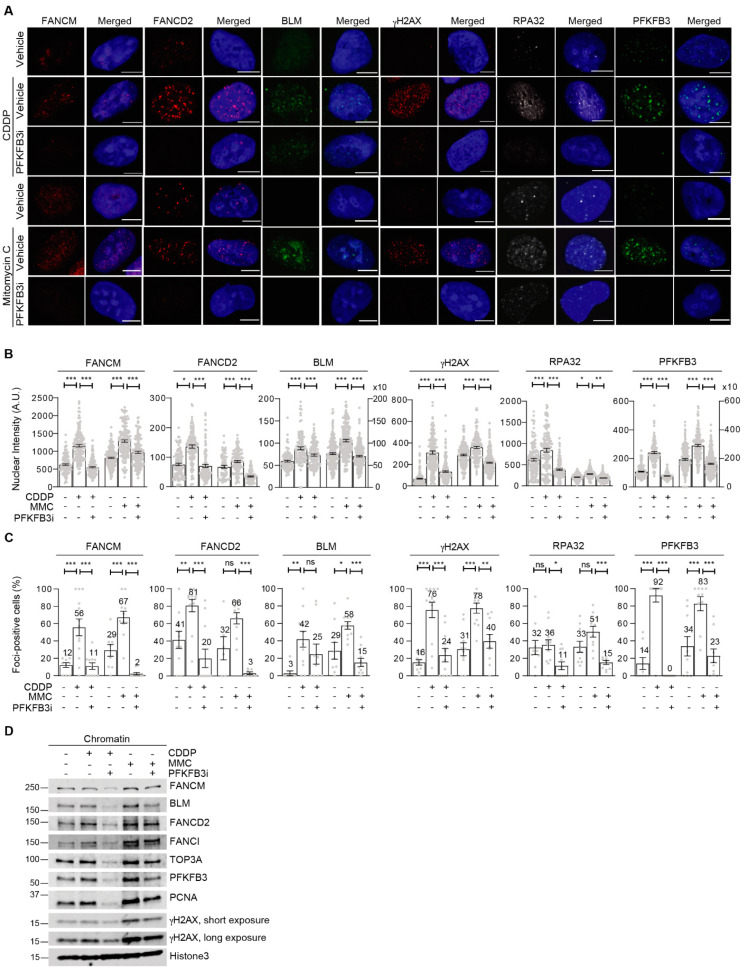
PFKFB3 kinase activity promotes recruitment of FA factors into foci. (**A**) Representative confocal images of nuclear foci of FA-associated factors FANCM, FANCD2, and BLM helicase, DNA damage surrogate marker γH2AX and RPA32 as well as PFKFB3, in synchronized U2OS cells exposed for 3 h to cisplatin (CDDP, 20 µM), Mitomycin C (MMC, 360 nM) and PFKFB3i (10 µM), or left untreated, *n* ≥ 2 independent experiments. Scale bar, 10 μm. (**B**) Quantification of FANCM, FANCD2, BLM, γH2AX, and PFKFB3 nuclear integrated intensity from (**A**). Quantification performed with CellProfiler. Data displayed as scatter dot plot with means ± S.E.M, one data point represents one cell, *n* ≥ 2 independent experiments, *n* > 100 cells per condition. * *p* < 0.05, *** *p* < 0.001; Unpaired two-tailed *t*-test between conditions. (**C**) Measurement of percentage of cells containing FANCM, FANCD2, BLM, γH2AX, and PFKFB3 foci number above the vehicle average from (**A**). Quantification executed using CellProfiler. Data represented as means ± S.E.M, one data point represents foci-positive percentage per picture, *n* ≥ 2 independent experiments, *n* > 100 cells per condition. * *p* < 0.05, ** *p* < 0.01, *** *p* < 0.001; Unpaired two-tailed *t*-test between conditions. (**D**) U2OS cells were treated with 6 µM aphidicolin for 24 h to allow synchronization at the G1/S boundary followed by release into vehicle, cisplatin (CDDP, 10 µM), Mitomycin C (MMC, 360 nM) and PFKFB3 inhibitor (10 µM) for 3 h. Cells were then subjected to fractionation for isolation and separation of whole cell soluble of chromatin-bound proteins followed by immunoblot, *n* > *2*. Images of the uncropped western blots can be found in Figure S12.

The foci recruitment of PFKFB3 upon FA pathway activation by FANCM and ATR prompted our interest to explore the mechanistic role of PFKFB3 in the pathway. In S-phase enriched cells, PFKFB3i reduced cisplatin- and MMC-induced chromatin recruitment and foci assembly of the BLM helicase (Figure 4A–D, Figures S6a and S12), known to be recruited to stalled replication forks and essential for fork restart and replication recovery upon ICL lesions [46]. Similarly, the interacting BLM partner TopIIIα failed to be recruited (Figure 4D). In line with the role of BLM in promoting FANCM recruitment to stalled replication forks [6], the addition of PFKFB3i also abolished FANCM and RPA recruitment and foci induction upon cisplatin and MMC treatments (Figure 4A–D, Figures S6a and S12). Strikingly, both chromatin recruitment and foci formation of FANCD2 was completely abolished upon PFKFB3i, as well as recruitment of its interacting partner FANCI (Figure 4A–D and Figure S12). Consequently, recruitment of PCNA, which is required for the recruitment and regulation of crosslink repair-coupled translesion synthesis, and γH2AX, a marker of DSB induction upon incision, were blocked upon PFKFB3i thus indicating defective ICL-repair downstream of FANCD2 (Figure 4A–D, Figures S6a and S12). A significant loss of PFKFB3 recruitment to chromatin and into foci was confirmed in these conditions, following the same pattern as repair factors assessed. Overall, recruitment was stronger upon MMC in comparison to cisplatin (Figure 4D and Figure S12), which could explain why the wide loss of recruitment of PFKFB3 and replication-coupled repair factors was stronger in cisplatin-treated samples (lanes 2 and 3) compared to MMC-treated cells (lanes 4 and 5). This difference could be due to MMC inducing a higher degree (approximately 3-fold) of ICLs than cisplatin [47].

We further identified an interaction between endogenous chromatin-bound PFKFB3 with FANCD2 and γH2AX in a manner dependent on its own activity (Figures S6b and S13a,b). Notably, the interaction with FANCD2 was decreased upon ATR inhibition while the interaction with γH2AX was ATR-independent, suggesting ATR-dependent and independent functions of PFKFB3 at the chromatin. Whereas, no interaction between BLM and FANCD2, nor BLM and γH2AX, could be identified at the chromatin in these conditions (Figures S6b and S13).

We conclude that PFKFB3 associates to DNA damage sites marked by γH2AX induction where it recruits FANCD2 through direct interaction to trigger its assembly into foci and establish FA repair. Consequently, blocking PFKFB3 activity disrupts the recruitment of FA repair proteins upon ICL induction.

### 3.6. PFKFB3 Inhibition Impairs Replication Fork Restart upon ICL-Induction

**Figure 5:**
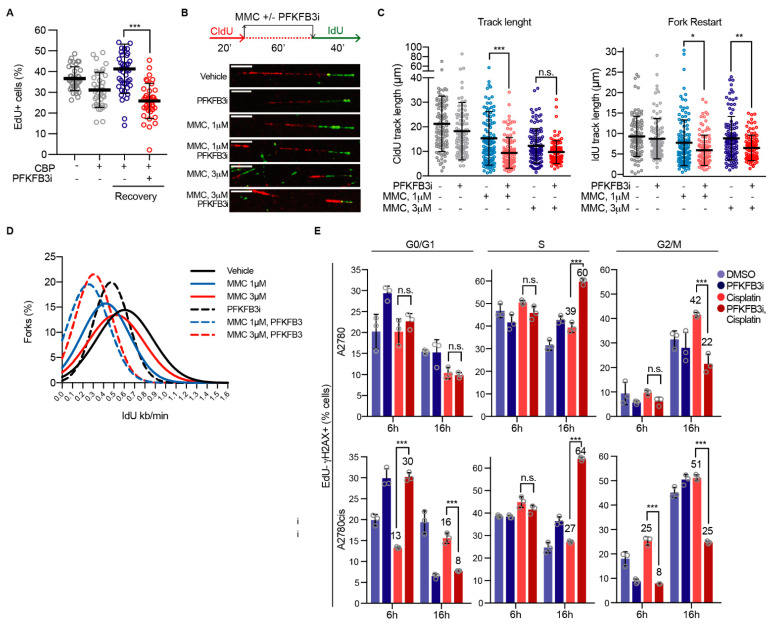
PFKFB3 inhibition impairs replication fork restart eliciting a FA-like phenotype in replicating cells. (**A**) Quantification of percentage of EdU positive A2780 cells. Cells were treated with carboplatin (CBP, 50 µM) for 20 h, followed by co-treatment with PFKFB3 inhibition (PFKFB3i, 10 µM) for 4 h. Cells were then released into normal media for 24 h, and pulse-labelled with 10 μM EdU for 30 min prior fixation. Cells were immunostained, subjected to confocal imaging, and EdU intensities were quantified using CellProfiler. EdU-positive threshold was established by the EdU intensity levels from the untreated sample. Data shown as means ± S.D., where each data point represents percentage of cells per picture, *n* = 2. *** *p* < 0.001; Mann–Whitney test. (**B**) A2780 cells were analyzed for replication fork restart. Shown is a schematic representation of the experimental setup and representative images of replication tracks for each condition. Cells were labeled with DNA analogs, CldU for 20 min followed by 60 min treatment with indicated concentrations of Mitomycin C (MMC) with or without PFKFB3 inhibition (B3i, 10 μM) and then IdU for 40 min. DNA fibers were prepared and visualized by immunofluorescence detection of CldU and IdU. Scale bar, 10 μm. (**C**) Fiber track length measurement from (**B**). Left panel shows mean CldU track lengths, right panel displays fork restart as mean IdU track lengths. One data point represents track length of a single fork, data are displayed as scatter dot plots with means ± S.D. of *n* > 100 forks per condition, representative experiment of *n* = 2, * *p*  < 0.05, ** *p*  < 0.01, *** *p* < 0.001; ordinary one-way ANOVA analysis, Tukey’s test for multiple comparisons. (**D**) Distribution of fork speed upon replication fork restart in cells in (**B**), *n* > 100 restarted forks per condition, representative experiment of *n* = 2. (**E**) Cell cycle analysis of EdU-γH2AX double positive A2780 and A2780cis cells. After cell cycle synchronization with aphidicolin (6 µM) for 24 h, cells were pulsed with EdU for 30 min and then released into cisplatin (10 µM) with or without PFKFB3i (5 µM) or left untreated for either 6 h or 16 h prior fixation. Data displayed as means ± SD of *n* > 20,000 events, representative experiment of *n* = 2, *** *p* < 0.001; ordinary one-way ANOVA analysis, Tukey’s test for multiple comparisons.

The impairment in recruitment of FA repair factors upon PFKFB3i prompted our interest to explore the functional consequences of inhibiting PFKFB3 upon ICL-induction during DNA replication. We first assessed replication dynamics upon addition of PFKFB3i to carboplatin-treated cells by quantifying the incorporation of 5-ethynyl-2′-deoxyuridine (EdU), a thymidine analogue. As expected, platinum treatment for 24 h led to a reduction in replicating cells, which resumed replication to vehicle levels after 24 h of recovery (Figure 5A). Notably, addition of PFKFB3i during the last 4 h of the total 24 h of carboplatin treatment followed by 24 h recovery significantly decreased the percentage of replicating cells. Considering the incapacity of cells to resume DNA synthesis upon PFKFB3i, we next monitored ICL-induced replication fork stalling and restarted on the single molecule level. We performed a dual-labeling protocol of the replicating DNA in which we first labelled replication tracks with the nucleoside analogue 5-Chloro-2′-deoxyuridine (CldU) together with MMC to stall replication forks in the presence or absence of PFKFB3i, followed by the release into 5-Iododeoxyuridine (IdU) to assess fork restart (Figure 5B). Already after 1 h treatment, PFKFB3i in combination with MMC significantly impeded replication fork progression and decreased CldU track lengths in comparison to MMC single treatments, whereas PFKFB3i on its own only modestly decreased track lengths in these experimental conditions (Figure 5B,C and Figure S7a). The percentage of stalled forks upon MMC treatments did not further increase upon PFKFB3 inhibition (Figure S7b), indicating that the observed decrease in CldU track lengths were due to degradation of MMC-stalled replication forks. Moreover, PFKFB3i resulted in shorter IdU track lengths and decreased fork speed upon restart in comparison to MMC-single treatments, as seen in the shift of distribution of fork speed (Figure 5C–E and Figure S7a), suggesting impaired fork restart. Moreover, post PFKFB3i and MMC combination treatments, fewer new origins fired were detected (second labelling with IdU, Figure S7c), suggesting a decrease in fork abundance.

Excessive degradation of MMC-stalled forks upon PFKFB3 inhibition may reduce fork stability, triggering fork collapse and the induction of DSBs. To investigate this further, we assessed the progression of replicating cells in relation to DNA damage induction. Cell synchronization at the G1/S-phase border was performed by aphidicolin treatment for 24 h, followed by release and EdU pulse labeling prior to adding the indicated drugs, which enabled the monitoring of cell cycle progression and DNA damage levels of cells actively replicating. In contrast to single treatments, PFKFB3i together with cisplatin resulted in a build-up of EdU positive cells with DNA damage (EdU-γH2AX double-positive) in the S-phase at 16 h, with cell population percentages increasing from 39% to 60%, and from 27% to 64%, in A2780 and A2780cis resistant cells, respectively (Figure 5E). Whereas, the EdU-γH2AX positive cell population increased in the G2/M phase upon cisplatin single treatments at 16 h (A2780) and 6 h (A2780cis) in comparison to vehicle. Notably, at 6 h treatment in A2780cis, PFKFB3i on its own increased the amount of EdU-γH2AX positive cells by 10% in the G0/G1 phase which may contribute to some extent to the accumulation of cells with DNA damage in the S-phase at 16 h. An overall enhanced level of DNA damage was confirmed by confocal analysis of γH2AX foci in cells synchronized and released for 16 h in the corresponding treatment (Figure S7d). Altogether, PFKFB3i results in a defective replication-fork recovery in sites of active replication post ICL-induction by MMC, ultimately resulting in an inability to replicate both cells to progress through the cell cycle and fork collapse, as assessed by the increased levels of EdU-γH2AX.

**Figure 6:**
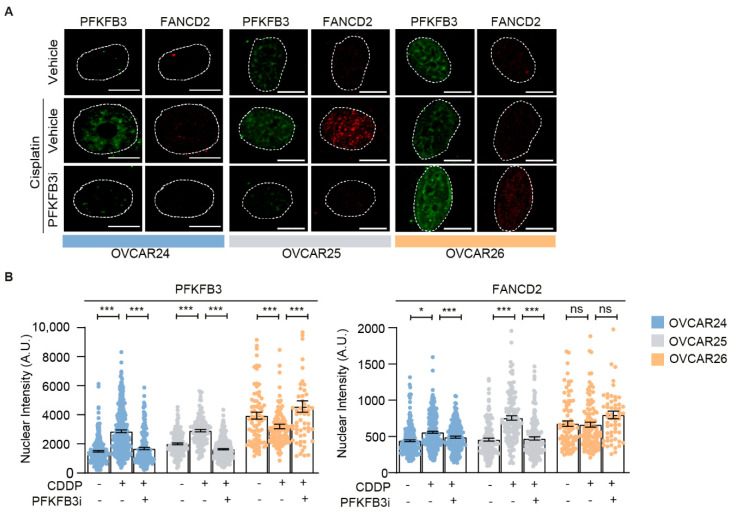
PFKFB3i treatment-induced FA repair defects in ex vivo cell models. (**A**) Representative confocal images of PFKFB3 and FANCD2 nuclear foci in patient-derived ovarian tumor cells, OVCAR24, OVCAR25 and OVCAR26, subjected to cisplatin (CDDP, 10 µM) treatment for 24 h in combination with PFKFB3i (10 µM), or left untreated. *n* = 2, scale bar = 10 μm. (**B**) Single cell analysis of PFKFB3 and FANCD2 nuclear intensity from (**A**). Data displayed as scatter plot with means ± S.E.M, *n* = 2 independent experiments, *n* > 100 cells per condition. * *p*  < 0.05, *** *p* < 0.001; unpaired two-tailed *t*-test between conditions.

Lastly, we validated our findings regarding the importance of PFKFB3 activity for functional FA repair in ex vivo cultures established from ovarian cancer patients. Importantly, in samples from two patients with functional FA pathway, as assessed by FANCD2 foci induction upon cisplatin, we could confirm the induction of PFKFB3 foci upon cisplatin as well as the loss of both cisplatin-induced FANCD2 and PFKFB3 foci upon PFKFB3i (OVCAR24 and OVCAR25 cells; Figure 6A,B). On the contrary, samples from a patient without cisplatin-induced FANCD2 foci, indicative of defective FA repair, did not display any induction of PFKFB3 foci (OVCAR26 cells; Figure 6A,B). Thus, PFKFB3 assembles into foci and regulates FA repair in vivo.

## 4. Discussion

Here, we provide the first evidence of PFKFB3 as a regulatory factor in replicative FA repair and its significance for functional FA repair upon DNA crosslink insults. Upon treatment with ICL inducing agents, ATR triggers relocation of PFKFB3 into foci where its presence is key for the recruitment of essential FA repair factors to establish functional FA repair, stabilization of stalled replication forks, and restoration of replication. Consequently, loss of PFKFB3 activity sensitizes cells to FA pathway activating drugs and reverses platinum resistance, known to be driven by FA pathway activation [41,42,43,44,48].

The key role of PFKFB3 in the FA pathway was noticeable andPFKFB3 inhibition reduced chromatin association of the FANCD2-FANCI complex and abolished FANCD2 foci formation (Figure 4, Figure 6 and Figure S6a,b), both consistent with the idea that the FANCD2-FANCI complex needs to be associated to DNA to be monoubiquitinated [13]; this posttranslational modification is required for FANCD2 foci formation [14]. Future studies should investigate if PFKFB3 inhibition indeed abolishes FANCD2 monoubiquitination directly through blocking its association with chromatin. Moreover, the recruitment of nuclear PFKFB3 was enhanced at concentrations of fork stalling agents reported to activate the FA pathway [40] (Figure 2C,D and Figure S4b,c). There was a direct protein–protein interaction between PFKFB3-FANCD2 and PFKFB3-γH2AX at the chromatin upon MMC treatments and, in addition, BLM and γH2AX foci formation and chromatin recruitment were dependent on PFKFB3 activity (Figure S6b). Consistent with the idea that BLM supports fork restart upon replicative stress through replication fork regression [46,49], that FANCD2-FANCI protects stalled forks at ICLs [13,50], and that FANCD2 suppresses MRE11-mediated fork degradation [51], PFKFB3i both impaired fork restart and decreased CldU track length without increasing percentage of stalled forks, indicative of fork degradation, upon MMC treatments (Figure 5A–D and Figure S7a,b). This resulted in substantial accumulation of replicating cells with DNA damage over time, an inability to progress out of S-phase, and reduced cell survival (Figure 1, Figure 2E and Figure 5E). Phenotypes resembling FA cells, due to their deficiency in ICL repair, accumulate at late S-phase upon ICL induction and display hypersensitivity to ICL-inducing agents [19,52].

In addition to stabilizing the ICL-stalled fork, FANCD2-FANCI are also required for the recruitment of nucleases for the incision step [19], which places PFKFB3 upstream of the nucleolytic cleavage of the stalled fork and DBS formation, arguing for a role of PFKFB3 in the FA pathway distinct from its role in HR repair [25,26]. In support of this, our findings place PFKFB3 upstream of MMC and cisplatin-induced γH2AX during early S-phase (3 h, Figure 4 and Figure S6a,b), whereas, upon DSB induction, PFKFB3 is downstream of γH2AX and dependent on γH2AX for relocation into foci to establish HR repair via the recruitment of RPA and RAD51 [25]. The following increase in percentage of EdU-yH2AX double positive cells in S-phase post-PFKFB3i and cisplatin combination treatments (Figure 5E) suggests potential fork collapse due to excessive degradation of MMC-stalled forks (Figure 5B,C and Figure S7). Furthermore, given that extensive stretches of ssDNA is not generated upon ICLs [12], the suppression of the modest increase in ICL-induced RPA foci formation upon PFKFB3i (Figure 4) may thus be distinct from the PFKFB3-mediated regulation of RPA assembly during HR which is linked to DSB resection [25]. Instead, we propose a model where the lack of FANCM foci is more likely to be the reason behind the lack of RPA foci [12].

PFKFB3 foci formation and interaction with FANCD2 was dependent on ATR activity (Figure 3A,B and Figure S6b), consistent with the idea that ATR is required for FANCD2 foci formation [53]. In this context, ATR mediates global fork slowing [54] and drives fork restart in concert with FANCM and FANCD2 upon ICL induction [55]. We could not, however, identify a direct interaction between PFKFB3 and FANCM. Upon cross-link induction, BLM interacts with mono-ubiquitinated FANCD2, although loss of BLM does not affect FANCD2 recruitment to the chromatin [56], consistent with the idea that FANCM interacts with BLM in an ATR-dependent manner independent of the FA core complex [6]. Since BLM associates with the FA core complex [7,8] and the loss of its helicase activity blocks FANCM recruitment and failure to perform ICL repair [6], the suppression of FANCM foci formation upon PFKFB3i might be a secondary effect due to loss of BLM (Figure 4 and Figure S6a). The dependence on FANCM for PFKFB3 recruitment (Figure 3) might thus reflect the reciprocal regulation of recruitment between FANCM and BLM to stalled forks [6,7]. In addition, BLM helicase activity supports fork restart upon replicative stress through replication fork regression [46,49]. We thus propose a model where PFKFB3 participates in an ATR-dependent positive feedback loop to protect the ICL-stalled fork, establishes FA repair, and limits DNA synthesis.

Mechanisms described to drive platinum resistance include enhanced DNA repair capacity [57]. For example, restoration of functional FA pathway, as measured by FANCD2 mono-ubiquitination, has been associated with cisplatin resistance [41,42,43,44,48]. Accordingly, the increased chromatin loading of FANCD2 and FANCI found in A2780cis-resistant cells compared to their sensitive counterparts (Figure 2F) indicates a potential increase in DNA repair capacity. In addition, stalled fork protection mechanisms have been accounted to resist platinum-containing drugs by promoting replication fork stability and resolution of stalled forks [58]. This is the case of BRCA2, which contributes to fork stabilization, and RAD51, which mediates fork remodeling and reversal, independent from their HR function [18,54,59,60,61,62]. Likewise, the current study describes a novel function of PFKFB3 in stalled fork recovery upon ICL-induction (Figure 5), independent from its already described role in HR [25,26]. Our results suggest that PFKFB3 deprivation results in fork collapse, demonstrated by γH2AX accumulation in replicating cells, which highlights the essentiality of PFKFB3 presence at stalled forks.

Here, we identify a cancer-specific synthetic lethality between PFKFB3 and platinum drugs treatment including restoring sensitivity to platinum in resistant cell models, independent of p53 and BRCA2 mutational status [63] (Figure 1). Cisplatin has been reported to promote glycolysis via triggering cytoplasmic localization of PFKFB3 in a cervical cancer cell line [23]. Although we detected an increase in glycolytic enzymes, including PFKFB3, in the whole cell soluble fraction upon cisplatin in transformed cell lines, the molecular mechanism behind the synergistic effects between platinum and PFKFB3 presented here appear to not be attributed to the inhibition of glycolysis (Figure 2A and Figure S2), but instead to a novel role of PFKFB3 in FA repair, potentially indicating differential functions of PFKFB3 depending on the tissue of origin. Firstly, inhibition of glycolysis upstream of PFKFB3 by 2-DG did not result in a cancer-specific synthetic lethality with carboplatin (Figure 1E), in line with the previously reported non-glycolytic role of PFKFB3 in promoting cell proliferation from its nuclear localization [20]. Secondly, platinum treatments did not alter glycolytic rates compared to vehicle, and PFKFB3i blocked glycolysis to a similar extent across the cancer transformation series (Figure 2A). Thirdly, alterations of the glucose concentration in the physiological [64] and supraphysiological range did not influence the EC_50_ of carboplatin (Figure S2d), suggesting that cancer cells tested in this study are not dependent on glucose for cell growth during carboplatin treatments under experimental conditions tested. Lastly, while glycolysis is a cytoplasmic process, cisplatin triggered an enrichment of PFKFB3 at the chromatin which was a signature of tumorigenic transformation (Figure 2B). In line with a lack of association of other glycolytic enzymes assessed to the chromatin during tested conditions (Figure 2F), PFKFB3 is the only glycolytic enzyme present at the replication fork in chromatin proteome-wide studies [65]. Notably, PFKFB3 also displayed an increased enrichment to the chromatin and into foci upon platinum resistance (Figure 2F and Figure S4d). In this context, PFKFB3 chromatin association correlated with both the cancer-specific synergy and the increased synergy score in resistant versus sensitive cancer cells. Thus, the DNA-damage-induced PFKFB3 recruitment upon cancer transformation and platinum resistance could be to favor DNA repair as an adaptive mechanism. Future studies should address whether the loss of PFKFB3 at the chromatin upon PFKFB3i or direct inhibition of its activity modulates FA repair, or a combination of both. In consonance with these findings, we have consistently shown that treatment with ICL-inducing agents including platinum drugs and MMC triggers nuclear recruitment of PFKFB3 concomitantly with DNA damage induction, measured by γH2AX, and impaired fork restart.

## 5. Conclusions

Overcoming platinum resistance remains a major clinical challenge. Drug combination strategies should improve the efficacy of platinum treatments not only by selectively sensitizing tumor cells, but also by targeting rewired DNA repair mechanisms in resistant cells. The novel role of PFKFB3 in mediating repair upon ICL-inducing agents presented here brings the opportunity for a combination treatment that is synthetic lethal to cancer cells.

## Data Availability

Data is contained within the article or Supplementary Materials or are available from the authors upon reasonable request.

## Supplementary Materials

The following are available online at https://www.mdpi.com/article/10.3390/cancers13143604/s1, Figure S1: PFKFB3 inhibition and ablation results in sensitization to platinum, Figure S2: Platinum drugs do not induce a preference for glycolysis, Figure S3: Validation of PFKFB3 association to chromatin, Figure S4: Nuclear recruitment of PFKFB3 upon DNA damage induction by interstrand crosslinking agents, Figure S5: ATR and FANCM dependent PFKFB3 foci induction, Figure S6: PFKFB3 stimulates recruitment of FA repair factors and interacts with FANCD2 and γH2AX at the chromatin, Figure S7: Targeting PFKFB3 induces defects in fork progression upon platinum treatments leading to fork collapse, Figure S8: Uncropped western blots from Figure 2B, Figure S9: Uncropped western blots from Figure S3a,b, Figure S10: Uncropped western blots from Figure 2F, Figure S11: Uncropped western blots from Figure S5b,e, Figure S12: Uncropped western blots from Figure 4D, Figure S13: Uncropped western blots from Figure S6b,c, Figure S14: Uncropped western blots from Figure 1G, Table S1: Antibodies used in the study.
