# Supplementary material for: PFKFB3 Inhibition Sensitizes DNA Crosslinking Chemotherapies by Suppressing Fanconi Anemia Repair
Source: Cancers (Basel). 2021 Jul 18;13(14):3604. doi: 10.3390/cancers13143604 (PMC8306909; doi:10.3390/cancers13143604)
Supplement: Supplementary file 1 [file cancers-13-03604-s001.zip › cancers-1283034-SI.pdf]

# Supplementary Material:

# PFKFB3 inhibition sensitizes DNA crosslinking chemotherapies by suppressing Fanconi Anemia repair

**Anna Huguet Ninou[1,2], Jemina Lehto[1,2], Dimitrios Chioureas[1], Hannah Stigsdotter[1], Korbinian Schelzig[1], Emma Åkerlund[1], Greta Gudoityte[1], Ulrika Joneborg[3], Joseph Carlson[4,5], Jos Jonkers[6], Brinton Seashore-Ludlow[1] and Nina Marie Susanne Gustafsson[1*]**

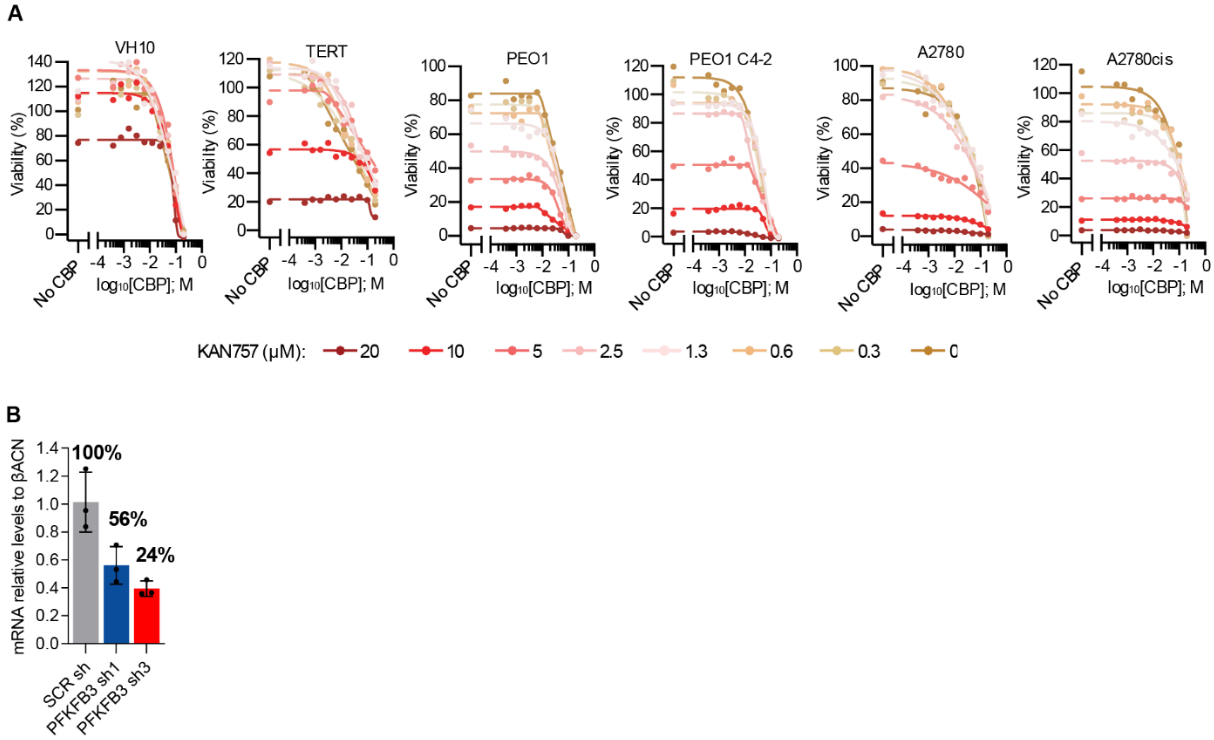


**Figure S1. Belongs to Figure 1. PFKFB3 inhibition and ablation results in sensitization to platinum therapeutics.** (A) Representative dose-response curves upon combination treatments of carboplatin and different doses of PFKFB3i (KAN757) for 72 h in BJhTERT and VH10 cells and the panel of EOC cells PEO1, PEO1-C4.2, A2780, Acis. Viability values were normalized to the untreated values. (B) RT-qPCR analysis of PFKFB3 expression levels normalized to β-actin mRNA expression levels (βACN) in A2780 cells transduced with shRNA targeting PFKFB3; data represent means ± S.D of $n$=3. Percentages indicate the relative PFKFB3 knock-down levels to shSCR.

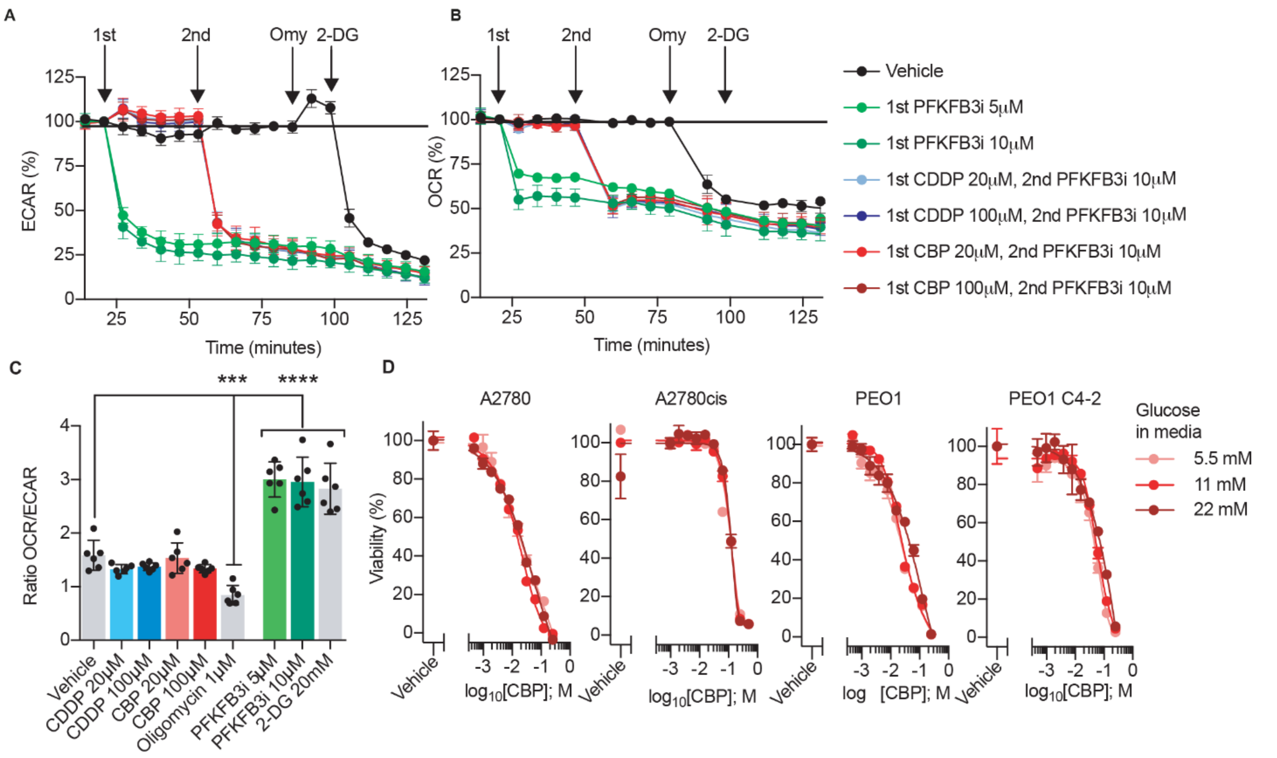


**Figure S2. Belongs to figure 2. Platinum drugs do not induce a preference for glycolysis.** (A) Extracellular Acidification Rate (ECAR) in A2780 cells. Cells were treated with indicated drugs and concentrations, all samples were treated with 2 μM Oligomycin (Omy) to obtain maximal ECAR capacity and subsequently with 20 mM 2-deoxy glucose (2-DG) for a complete block of glycolysis. Data displayed as average value at each time point ± S.D, *n*=6. (B) Baseline Oxidative Consumption Rate (OCR) in A2780 cells treated as (A). Data displayed as average value at each time point ± S.D, *n*=6. (C) Bars represent OCR/ECAR ratio calculated from (A) and (B). Average values used to calculate ratio from measurements at time point=33,7min; t=98 min for Oligomycin and t=118 min for 2-DG vehicle controls. Average values ± S.D, *n*=6. ***P<0.001, ****P<0.0001; One-way ANOVA analysis. (D) Carboplatin dose-response curves in media containing 5.5 mM, 11 mM or 22 mM glucose as evaluated by resazurin assays upon 72 h of treatment. Each viability value is displayed as means ± S.D, *n*=2, shown is a representative experiment.

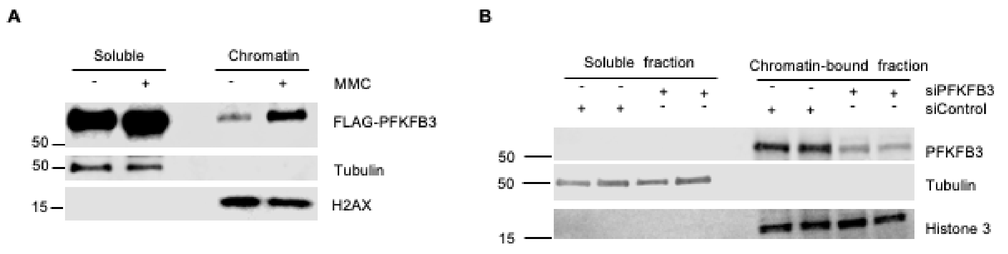


**Figure S3. Belongs to Figure 2. Validation of PFKFB3 association to chromatin.** (A) FLAG-PFKFB3 transfected U2OS cells were synchronized at the G1/S boundary and then treated with Mitomycin C (360 nM) for 3 h or left untreated. After harvesting, cells were subjected to cell fractionations for isolation of soluble and chromatin-bound proteins, followed by immunoprecipitations and immunoblot. (B) U2OS cells were transfected with siControl or siPFKFB3 for 24h nd subjected to cell fractionations for isolation of soluble and chromatin-bound proteins, followed by immunoprecipitations and immunoblot. Images of the uncropped Western blots can be found in Figure S9.

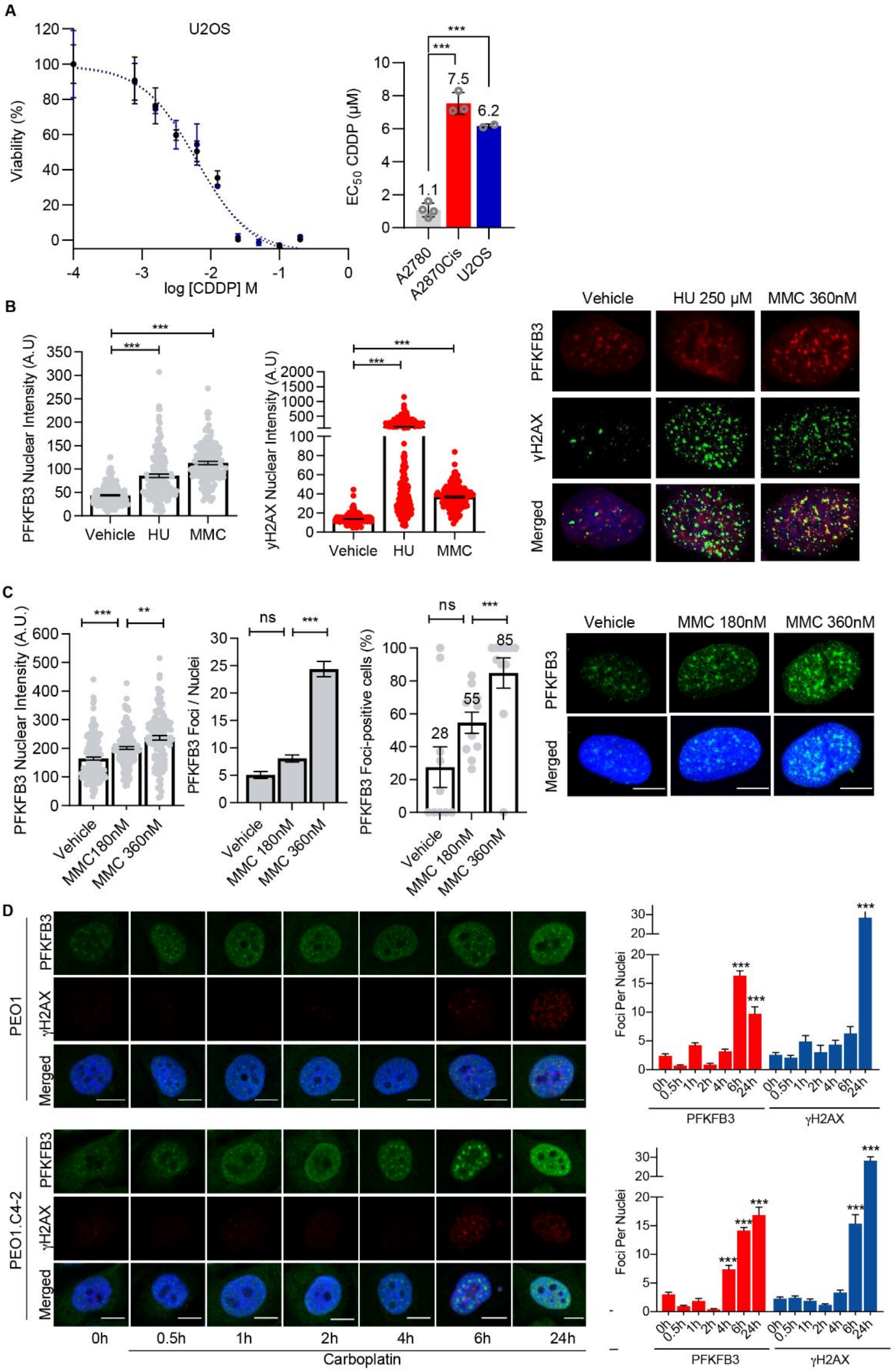


**Figure S4. Belongs to Figure 2. Nuclear recruitment of PFKFB3 upon DNA damage induction by interstrand crosslinking agents.** (A) Representative dose-response curves of U2OS cells upon elevating concentrations of cisplatin (CDDP) in 72 h resazurin viability assay. Viability values were normalized to untreated cells. Experiment was repeated twice. To the right, graph with cisplatin $EC_{50}$ values of U2OS, platinum sensitive A2780 and platinum resistant A2780Cis cells. ****P* > 0.0001; Unpaired t-test. (B) Confocal analysis of nuclear PFKFB3 intensity and DNA damage induction in U2OS treated for 24 h prior fixation with HU (250 µM), MMC (360 nM) or left untreated. Quantification of PFKFB3 (left) and γH2AX (middle) nuclear intensity using CellProfiler. Data displayed as average ± S.E.M, individual points represent measured intensity in single cells, *n>100* cells. **P<0.001; One-way ANOVA analysis for statistical significance, Dunnett's test for multiple comparisons. To the right, representative images. (C) Confocal analysis of nuclear PFKFB3 levels in U2OS cells treated with MMC (180 nM or 360 nM) for 3 h, or left untreated. Left graph shows scatter plot of PFKB3 nuclear intensity,

individual points represent intensity of single cells. The middle graph shows average number of PFKFB3 foci per nuclei, data shown as bar graph. The right graph shows the percentage of PFKFB3 foci-positive cells compared to the average foci per cell in vehicle sample, one data point represents foci-positive percentage per picture. Data is shown as means ± S.E.M. ***P<0.001; One-way ANOVA analysis. To the right, representative images, scale bar=10 μm. (D) γH2AX and PFKFB3 recruitment kinetics upon carboplatin treatment (CBP) in PEO1 (top) and PEO1.C4-2 (bottom) isogenic ovarian cancer cells. To the left, representative images of γH2AX and PFKFB3 foci induction through the time-course analysis. Scale bar, 10 μm. To the right, quantification of PFKFB3 and γH2AX foci per nuclei in PEO1 sensitive cells (top panel) and PEO1.C4-2 platinum-resistant cells (bottom panel). Data displayed as means ± S.E.M, representative of *n*=2 experiments, *n>100* cells per condition. ***P<0.0001; Ordinary one-way ANOVA, Sidak's test for multiple comparisons.

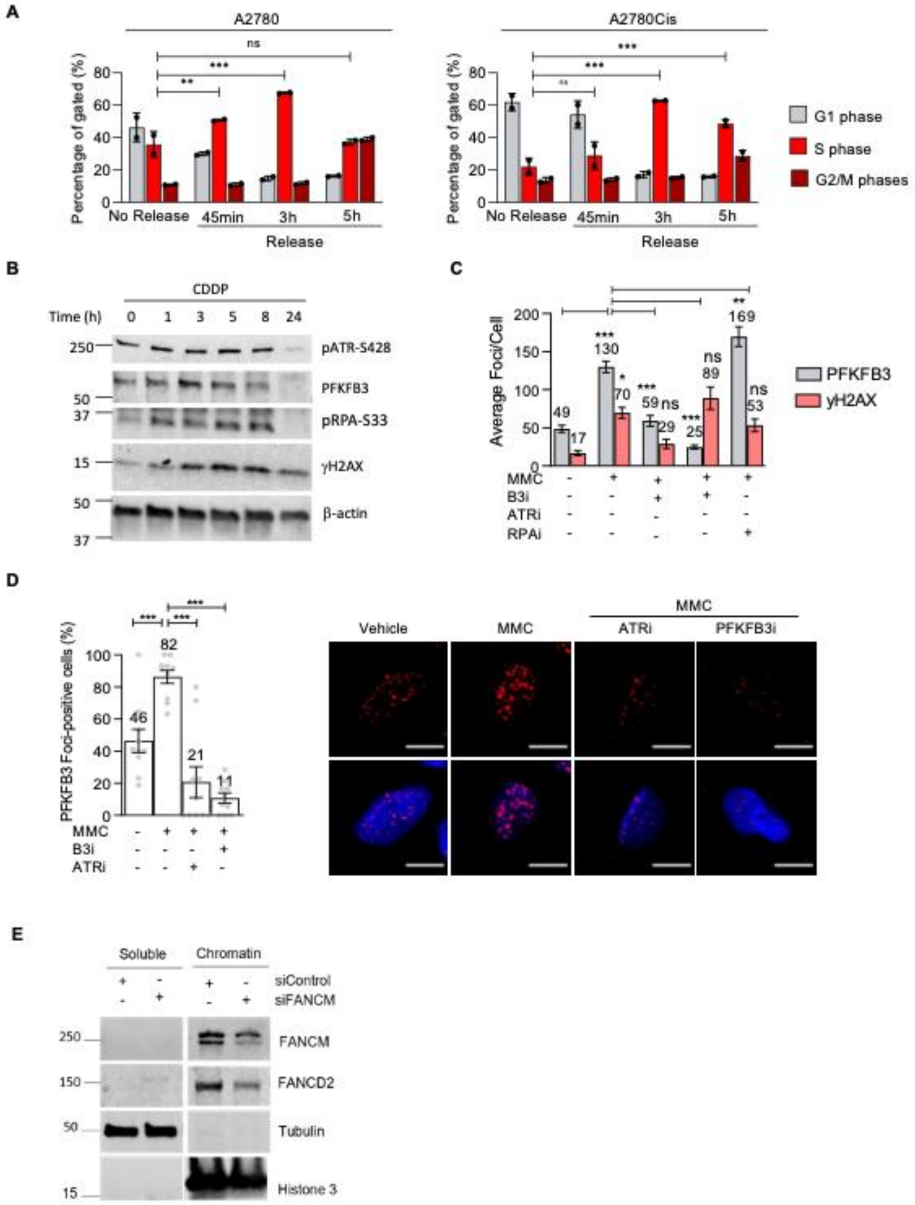


**Figure S5. Belongs to Figure 3. ATR and FANCM dependent PFKFB3 foci induction.** (A) DNA histograms of A2780 and A2780cis cells fixed at indicated times after cell cycle synchronization with aphidicolin (6 μM) for 24 h and release in normal media. Cells were fixed at different time points to determine kinetics of S-phase cell cycle progression based on DNA content, which was assessed by propidium iodide staining and flow cytometry. Data displayed as means ± SD of *n>20,000* events. ***P* < 0.01, ***P < 0.001; ordinary one-way ANOVA analysis, Tukey's test for multiple comparisons. (B) Immunoblot of A2780 cells harvested at indicated times after cell cycle synchronization with aphidicolin and released into cisplatin (CDDP, 20 μM) for indicated time-points. Total protein levels of DNA damage response proteins pATR-S428, pRPA-S33 and γH2AX correlated with induction of PFKFB3 protein levels. β-Actin was used as a loading control. Images of the uncropped Western blots can be found in Supplementary Figure 11. (C) Quantification of PFKFB3 and γH2AX average nuclear foci per cell in U2OS cells synchronized and release for 3 h in vehicle or Mitomycin C (MMC, 360nM) with or without indicated inhibitors. Image-based analysis was performed using CellProfiler. Data displayed as means ± S.E.M, *n=2* independent experiments, *n>100* cells per condition. *P < 0.1, **P < 0.01, ***P<0.001; Ordinary one-way ANOVA, Sidak's test for multiple comparisons. (D) Measurement of PFKFB3 foci-positive cells in U2OS cells exposed to Mitomycin C (MMC, 360 nM) together with PFKFB3i (10 μM) or ATRi (2.5 μM) for 3 h. Cells were seeded and next day were treated and fixed without cell cycle synchronization. Images were analysed using CellProfiler. Left panel depicts the percentage of cells containing number of PFKFB3 nuclear foci above the average in vehicle sample. Data displayed as means ± S.E.M, one data point represents foci-positive percentage per picture, *n>100* cells per condition, *n* = 2. ***P<0.001; Ordinary one-way ANOVA, Sidak's test for multiple comparisons. To the left, representative images of PFKFB3 nuclear foci. Scale bar, 10 μm. (E) U2OS cells were treated with either siRNA control or targeting FANCM for 24h, followed by treatment with 6 μM aphidicolin for 24h and release into cell media for 3h prior harvesting. Cells were subjected to fractionation for isolation of chromatin-bound proteins followed by immunoblot. Images of the uncropped Western blots can be found in Supplementary Figure 11.

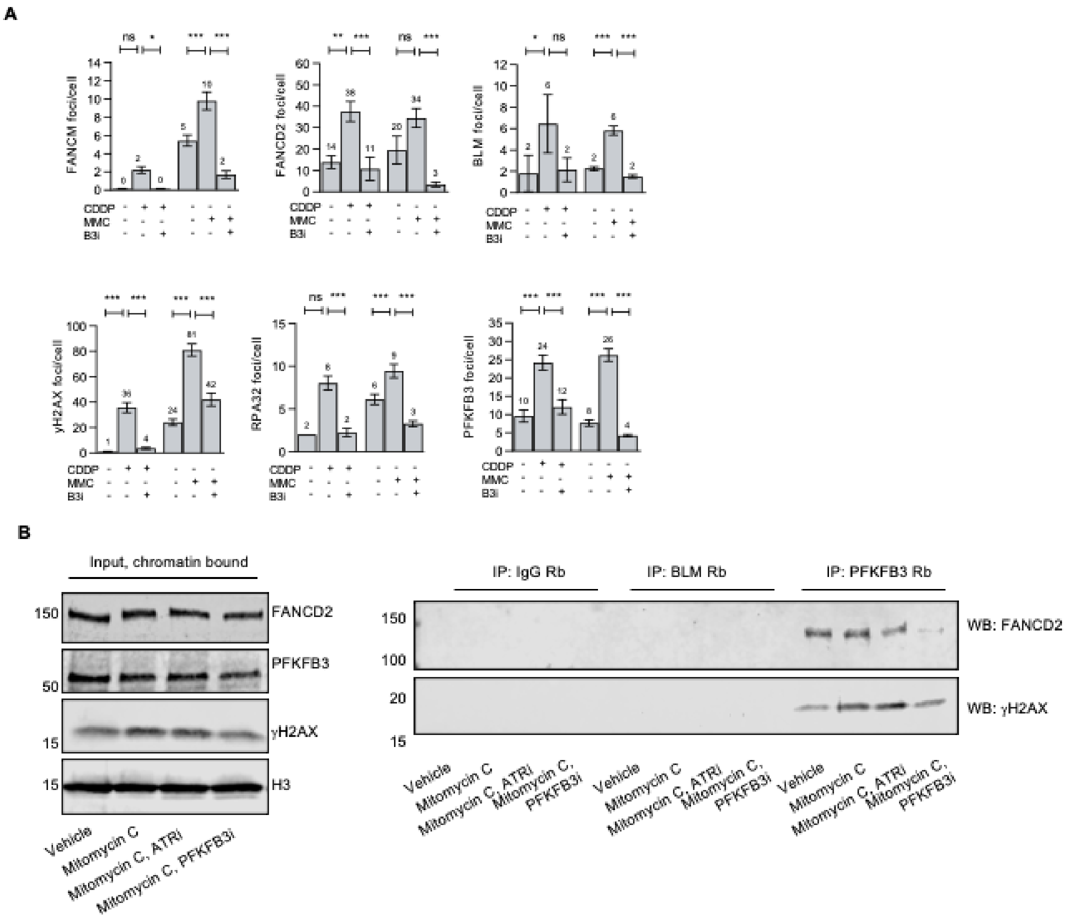


**Figure S6. Belongs to Figure 4.** PFKFB3 stimulates recruitment of FA repair factors and interacts with FANCD2 and γH2AX at the chromatin. (A) Quantification of foci per nuclei of FANCM, FANCD2 and BLM, γH2AX and PFKFB3 in Figure 4A. Data displayed as foci number means ± S.E.M, *n=2* independent experiments, *n>100* cells per condition. *P < 0.1, **P < 0.01, ***P<0.001; Ordinary one-way ANOVA, Sidak's test for multiple comparisons. (B) U2OS cells were synchronized at the G1/S boundary and then treated with Mitomycin C (360 nM) in combination with ATRi (2.5μM) or PFKFB3i (10μM for 3 h or left untreated. After harvesting, cells were subjected to cell fractionations for isolation of soluble and chromatin-bound proteins, followed by co-immunoprecipitations and immunoblot. To the left, input samples, histone 3 was used as loading control. To the right, immunoprecipitation of endogenous PFKFB3 and BLM. Images of the uncropped Western blots can be found in Supplementary Figure 13.

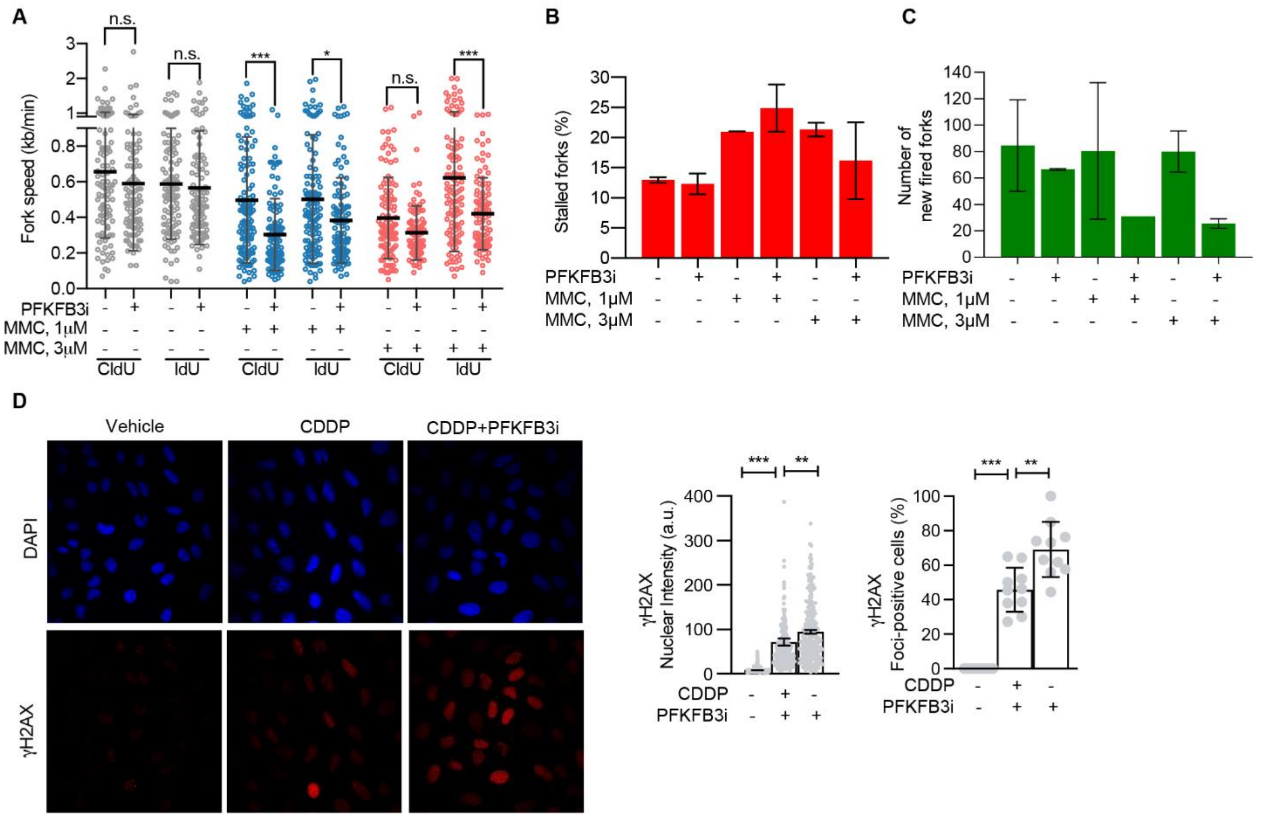


**Figure S7.** Belongs to Figure 5. Targeting PFKFB3 induces defects in fork progression upon platinum treatments leading to fork collapse. (A) Replication fork speed analysis from Figure 5B. One data point represents fork speed of single fork upon either CldU or IdU pulse as indicated. Data shown as means ± S.D. of *n>100* forks per condition, representative experiment of *n*=2, *$P < 0.05$, **$P < 0.01$, ***$P < 0.001$; ordinary one-way ANOVA analysis, Tukey's test for multiple comparisons (B) Percentage of stalled forks during the first pulse after indicated treatments (fibers only labeled with CIdU) in Figure 5B. Data is shown as means ± S.D of *n=2* independent replicates with *n>100* forks per condition. (C) Number of new origins fired during the second pulse after indicated treatments (fibers only labeled with IdU) in Figure 5B. Data is shown as means ± S.D of *n*=2 independent replicates. (D) Confocal analysis of DNA damage marker, γH2AX, in U2OS cells synchronized to G1/S cell cycle phase boundary and released into cisplatin (CDDP, 10 μM), cotreated with CDDP (10 μM) and PFKFB3i (10 μM), or left untreated for 16 h. To the left, representative images used for the analysis. Scale bar, 10 μm. To the right, graphs displaying γH2AX nuclear intensity, data displayed as means ± S.E.M, individual points represent intensity of single cells; and percentage of cells that contain above the cisplatin single treatment average number of γH2AX foci per cell. Data displayed as means ± S.E.M, representative of *n*=2 experiments, *n>100* cells per condition. **P<0.01, ***P<0.001; One-way ANOVA analysis, Dunnett's test for multiple comparisons.

**A**

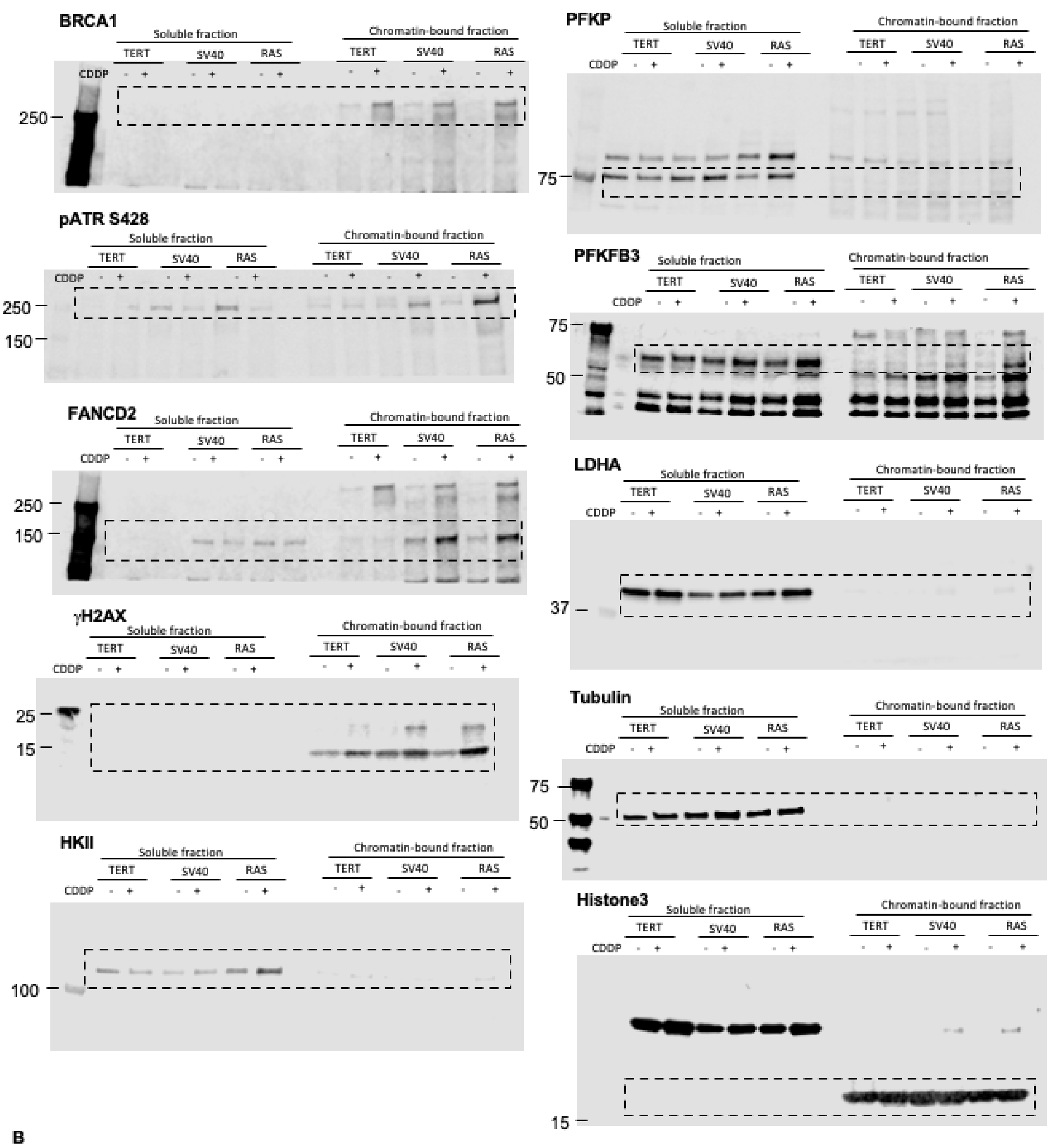


**B**

**Densitometry Readings, Figure 2B, Figure S8**

| | Normalised to Tubulin | | | | | | Normalised to Histone 3 | | | | | |
|---|---|---|---|---|---|---|---|---|---|---|---|---|
| **Lane:** | **1** | **2** | **3** | **4** | **5** | **6** | **7** | **8** | **9** | **10** | **11** | **12** |
| **BRCA1** | 0.000 | 0.000 | 0.000 | 0.000 | 0.000 | 0.000 | 0.688 | 5.705 | 0.107 | 2.742 | 0.028 | 3.327 |
| **p-ATRS428** | 0.000 | 0.004 | 0.005 | 0.000 | 0.010 | 0.002 | 0.022 | 0.021 | 0.032 | 0.082 | 0.019 | 0.202 |
| **FANCD2** | 0.000 | 0.000 | 0.000 | 0.000 | 0.000 | 0.000 | 0.312 | 1.926 | 0.245 | 1.175 | 0.066 | 1.692 |
| **γH2AX** | 0.000 | 0.000 | 0.000 | 0.000 | 0.000 | 0.000 | 0.742 | 0.116 | 0.106 | 0.144 | 0.075 | 0.173 |
| **HKII** | 0.044 | 0.025 | 0.019 | 0.016 | 0.034 | 0.046 | 0.086 | 0.095 | 0.064 | 0.082 | 0.038 | 0.087 |
| **PFKP** | 0.051 | 0.032 | 0.036 | 0.031 | 0.021 | 0.035 | 0.011 | 0.011 | 0.011 | 0.010 | 0.009 | 0.010 |
| **PFKFB3** | 0.003 | 0.035 | 0.027 | 0.019 | 0.020 | 0.046 | 0.022 | 0.021 | 0.032 | 0.062 | 0.009 | 0.163 |
| **LDHA** | 0.420 | 0.328 | 0.109 | 0.099 | 0.157 | 0.224 | 0.075 | 0.053 | 0.021 | 0.093 | 0.028 | 0.111 |

**Figure S8. Uncropped western blots from Figure 2b.** (**a**) Uncropped western blot membranes, dotted lines indicate the cropped area used in Figure 2b western blot. (**b**) Quantification of protein expression from (**a**) measured by Western blot densitometry, normalized to tubulin for soluble fractions and histone 3 for chromatin bound fractions.

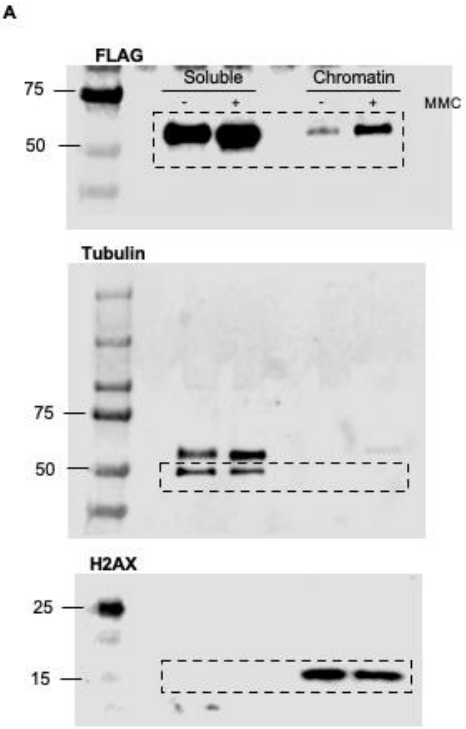


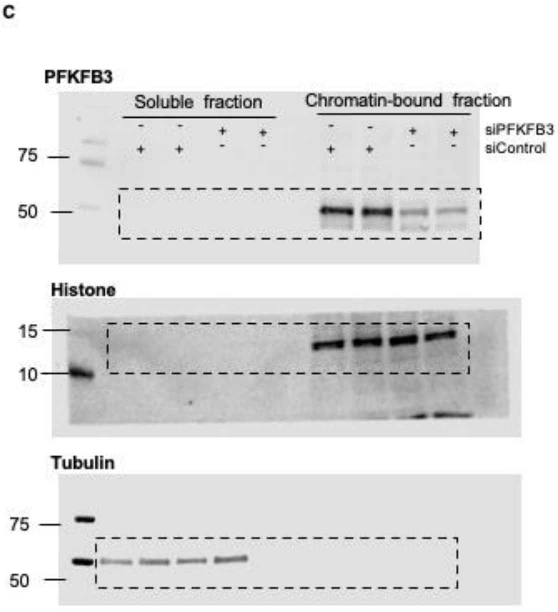


B

**Densitometry Readings, Figure S3a, S9a**

| Lane: | 1 | 2 | 3 | 4 |
|---|---|---|---|---|
| FLAG | 10.7 | 16.2 | 0.178 | 1.37 |
| Tubulin | 8.89 | 6.44 | | |
| H2AX | 0 | 0 | 0.668 | 0.712 |

D

**Densitometry Readings, Figure S3b, S9c**

| Lane: | 1 | 2 | 3 | 4 |
|---|---|---|---|---|
| PFKFB3 | 9.76 | 14.8 | 3.02 | 2.71 |
| H3 | 0.17 | 0.18 | 0.21 | 0.16 |

**Figure S9. Uncropped western blots from Figure S3a, b.** (**a**) Uncropped western blot membranes, dotted lines indicate the cropped area used in Figure S3a western blot. (**b**) Quantification of protein expression from (**a**) measured by Western blot densitometry, normalized to tubulin for soluble fractions and histone 3 for chromatin bound fractions. (**c**) Uncropped western blot membranes, dotted lines indicate the cut sections used in Figure S3b western blot. (**d**) Quantification of protein expression from (**c**) measured by Western blot densitometry.

A

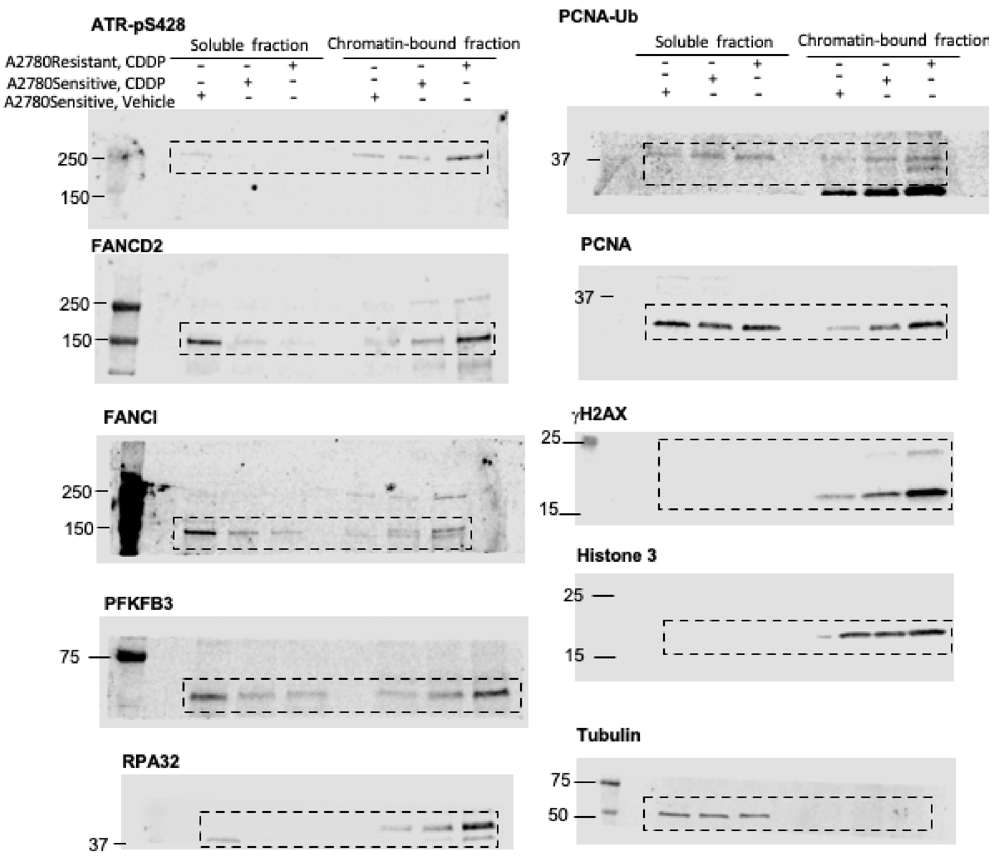


B

Densitometry Readings, Figure 2f, Figure S10

| | Normalised to Tubulin | | | Normalised to Histone 3 | | |
|---|---|---|---|---|---|---|
| **Lane:** | **1** | **2** | **3** | **4** | **5** | **6** |
| **p-ATRS428** | 0.00395257 | 0 | 0 | 0.00347222 | 0.00346021 | 0.08287293 |
| **FANCD2** | 0.02371542 | 0.00269542 | 0 | 0.00347222 | 0.00692042 | 0.01933702 |
| **FANCI** | 0.01581028 | 0.0032345 | 0.00478469 | 0 | 0.00346021 | 0.00828729 |
| **PFKFB3** | 0.02766798 | 0.00539084 | 0.00478469 | 0.00694444 | 0.01384083 | 0.02209945 |
| **RPA32** | 0.01976285 | 0.00269542 | 0.00478469 | 0.01388889 | 0.02768166 | 0.06353591 |
| **PCNA UbL164** | 0.00395257 | 0.00269542 | 0.00478469 | 0 | 0.00346021 | 0.00552486 |
| **PCNA** | 0.0513834 | 0.0296496 | 0.00669856 | 0.13888889 | 0.27681661 | 0.0441989 |
| **γH2AX** | 0 | 0 | 0 | 0.02430556 | 0.04844291 | 0.12707182 |

**Figure S10. Uncropped western blots from Figure 2f.** (**a**) Uncropped western blot membranes, dotted lines indicate the cropped area used in Figure 2f western blot. (**b**) Quantification of protein expression from (**a**) measured by Western blot densitometry, normalized to tubulin for soluble fractions and histone 3 for chromatin bound fractions.

A

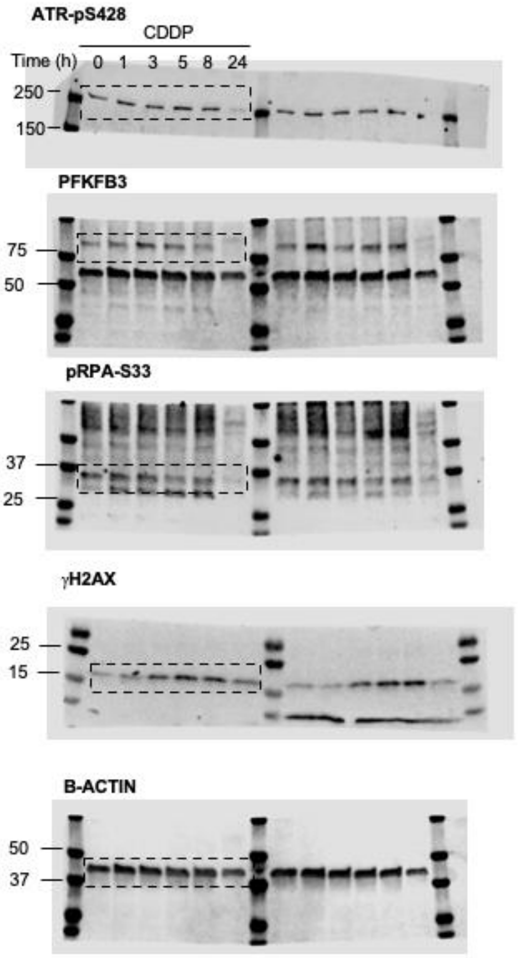


B

Densitometry Readings, Figure S5b

Normalised to Tubulin

| Lane: | 1 | 2 | 3 | 4 | 5 | 6 |
|---|---|---|---|---|---|---|
| p-ATRS428 | 0.01 | 0.01 | 0.01 | 0.01 | 0.01 | 0 |
| PFKFB3 | 0.20 | 0.23 | 0.32 | 0.26 | 0.20 | 0.05 |
| pRPA-S33 | 0.02 | 0.03 | 0.02 | 0.03 | 0.04 | 0 |
| γH2AX | 0.01 | 0.02 | 0.02 | 0.01 | 0.04 | 0.04 |

C

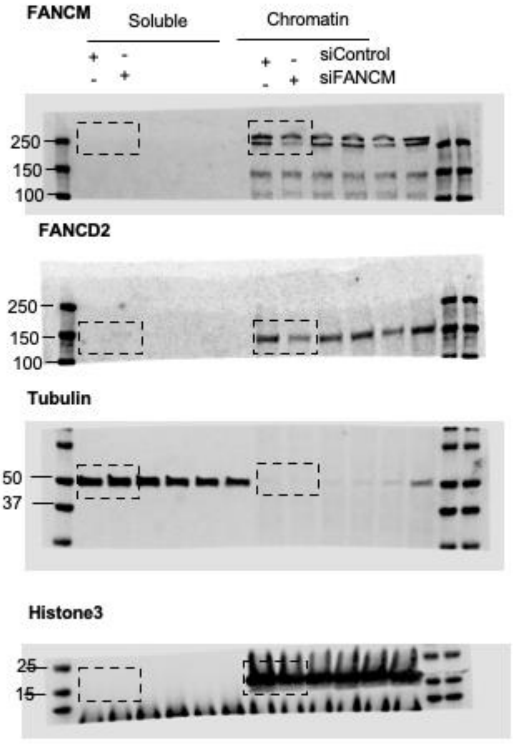


D

Densitometry Readings, Figure S5e

Lane 1, 2 normalised to Tubulin;

Lane 3, 4 normalised to Histone 3

| Lane: | 1 | 2 | 3 | 4 |
|---|---|---|---|---|
| FANCM | 0 | 0 | 0.0067 | 0.0042 |
| FANCD2 | 0 | 0.004 | 0.1326 | 0.0568 |

**Figure S11. Uncropped western blots from Figure S5b, e.** (**a**) Uncropped western blot membranes, dotted lines indicate the cropped area used in Figure S5b western blot. (**b**) Quantification of protein expression from (**a**) measured by Western blot densitometry, normalized to tubulin. (**c**) Uncropped western blot membranes, dotted lines indicate the cropped area used in Figure S5e western blot. (**d**) Quantification of protein expression from (**c**) measured by Western blot densitometry, normalized to tubulin.

A

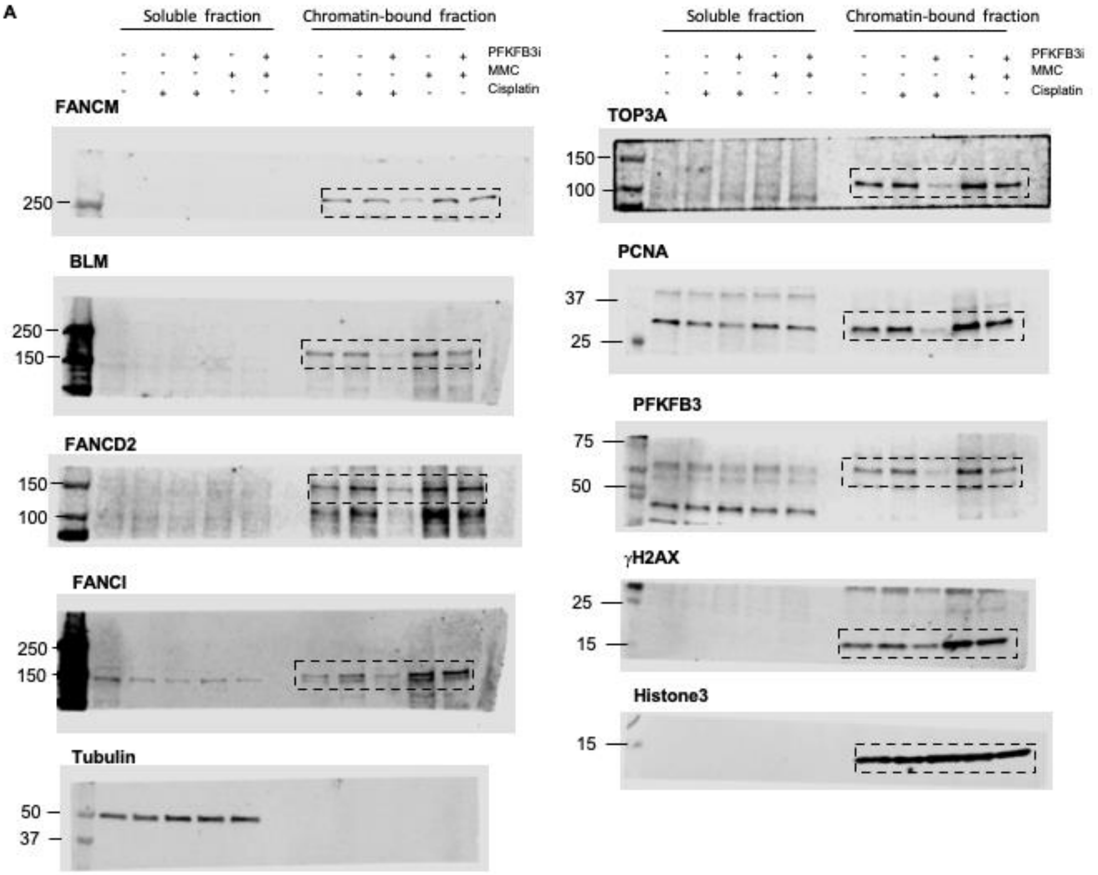


B

**Densitometry Readings, Figure 4d, Figure S12**

**Normalised to Histone 3**

| Lane: | 1 | 2 | 3 | 4 | 5 |
|---|---|---|---|---|---|
| FANCM | 0.08298755 | 0.12345679 | 0.03039514 | 0.16891892 | 0.14981273 |
| BLM | 0.24896266 | 0.32921811 | 0.03039514 | 0.40540541 | 0.26217228 |
| FANCD2 | 0.08298755 | 0.16460905 | 0.03039514 | 0.16891892 | 0.2247191 |
| FANCI | 0.24896266 | 0.41152263 | 0.09118541 | 0.60810811 | 0.37453184 |
| TOP3A | 0.58091286 | 0.69958848 | 0.12158055 | 0.81081081 | 0.48689139 |
| PFKFB3 | 2.54273859 | 2.94238683 | 0.47720365 | 3.47297297 | 1.87940075 |
| PCNA | 0.62240664 | 0.82304527 | 0.06079027 | 1.4527027 | 0.88014981 |
| γH2AX | 0.82987552 | 0.82304527 | 0.39513678 | 1.68918919 | 1.16104869 |

**Figure S12. Uncropped western blots from Figure 4d.** (**a**) Uncropped western blot membranes, dotted lines indicate the cropped area used in Figure 4d western blot. (**b**) Quantification of protein expression from (**a**) measured by Western blot densitometry, normalized to histone 3.

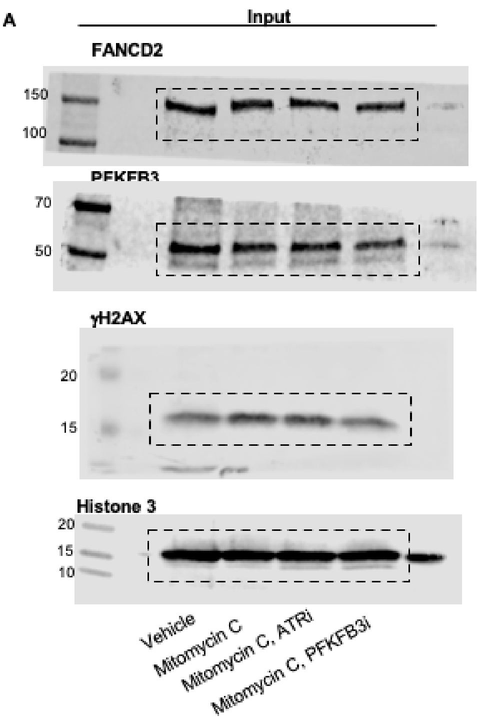


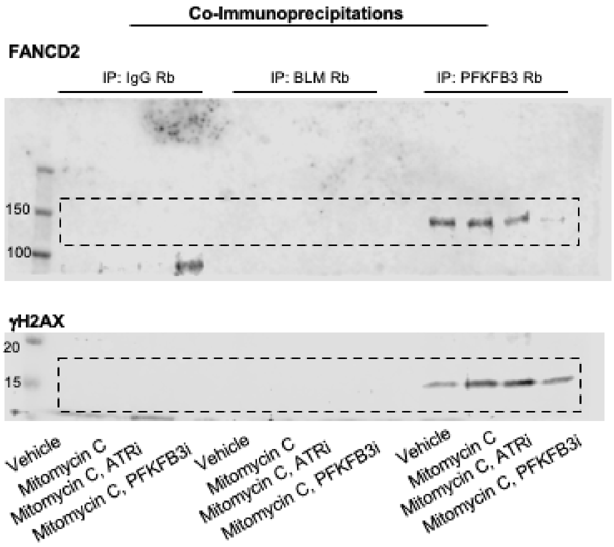


B

**Densitometry Readings, Figure S6b, Figure S13**

| 6B Input, lane: | 1 | 2 | 3 | 4 |
|---|---|---|---|---|
| FANCD2 | 0.49 | 0.41 | 0.44 | 0.4 |
| PFKFB3 | 3.27 | 2.25 | 2.18 | 1.77 |

| 6B IP, lane | 1 | 2 | 3 | 4 |
|---|---|---|---|---|
| FANCD2 | 0.06 | 0.8 | 0.05 | 0.03 |
| γH2AX | 0.77 | 1.11 | 1.05 | 0.67 |

**Figure S13. Uncropped western blots from Figure S6b,c.** (**a**) Uncropped western blot membranes, dotted lines indicate the cropped area used in Figure S6b western blot. (**b**) Quantification of protein expression from (**a**) measured by Western blot densitometry.

PFKFB3

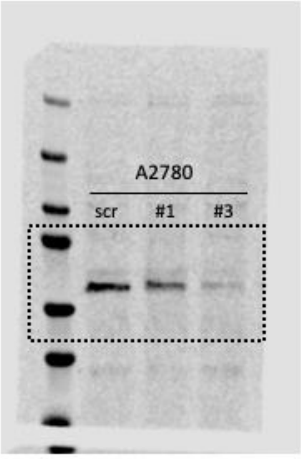


β-actin

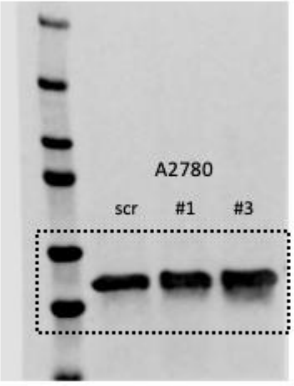


**Densitometry Readings, Figure 1G, Figure S14**

**Normalised to β-Actin**

| Lane | 1 | 2 | 3 |
|---|---|---|---|
| PFKFB3 | 1 | 0.61 | 0.31 |

**Figure S14. Uncropped western blots from Figure 1g.** Uncropped western blot membranes, dotted lines indicate the cropped area used in Figure 1g western blot. Table shows quantification of protein expression from measured by Western blot densitometry.

**Table S1.** Antibodies used in the study.

| Antibody | Source | Catalog # | Dilution, application |
|---|---|---|---|
| PFKFB3 | Proteintech | 13763-1-AP | 1:100, IF; 1:400, WB |
| PFKFB3 (D7H4Q) | Cell Signaling | 13123 | 1:500, WB |
| HK II (C64G5) | Cell Signaling | 2867 | 1:500, WB |
| PFKFP (D4B2) | Cell Signaling | 8164 | 1:500, WB |
| LDHA (C4B5) | Cell Signaling | 3582 | 1:500, WB |
| p-H2A.X S139 | Cell Signaling | 2577S | 1:1000, WB |
| p-H2A.X S139 | Millipore | 05-636 | 1:500, IF and flow cytometry |
| H2AX | Abcam | ab18255 | 1:2000,WB |
| $\alpha$-tubulin | Abcam | ab18251 | 1:10,000, WB |
| β-actin | Abcam | ab6276 | 1:10,000, WB |
| Histone 3 | Abcam | Ab1791 | 1:5000, WB |
| BRCA1 (D-9) | Santa Cruz | sc6954 | 1:200, WB |
| p-ATR S428 | Cell Signaling | 2853S | 1:500, WB |
| FANCD2 (FI17) | Santa Cruz | sc-20022 | 1:200, WB; 1:200 IF |
| FANCI (A-7) | Santa Cruz | sc-271316 | 1:200, WB |
| FANCM | Novus Biologicals | NBP2-50418 | 1:200, WB ; IF 1:100 |
| FLAG | Sigma Aldrich | F3165 | 1:5000, WB; 2μg, IP |
| BLM | Bethyl Laboratories | A300-110A-M | 1:200, WB; 1:100, IF |
| RPA32 | Cell Signaling | 2208S | 1:500, WB; 1:200, IF |
| p-RPA-S33 | Novus Biological | NB100-544 | 1:500, WB |
| PCNA (F-2) | Santa Cruz | sc25280 | 1:400, WB |
| Ubiquityl-PCNA (Lys164) | Cell Signaling | 13439 | 1:400, WB |
| Topoisomerase III | Santa Cruz | sc11257 | 1:200, WB |
| IRDye 680RD Donkey anti-Mouse IgG | Li-Cor Biosciences | 925-68072 | 1:5000, WB |
| IRDye 800CW Donkey anti-Rabbit IgG | Li-Cor Biosciences | 925-32213 | 1:5000, WB |
| RDye 680CW Donkey anti-Rabbit IgG | Li-Cor Biosciences | 925-68073 | 1:5000, WB |
| IRDye 800CW Donkey anti-Mouse IgG | Li-Cor Biosciences | 926-32212 | 1:5000, WB |
| IRDye 800CW Donkey anti-Rat lgG | Li-Cor Biosciences | 926-32219 | 1:5000, WB |
| IFDye 800CW Donkey anti-Goat lgG | Li-Cor Biosciences | 926-32214 | 1:5000, WB |
| Donkey anti-mouse Alexa Fluor 488 | Invitrogen | A-21202 | 1:500, IF |
| Donkey anti-rabbit Alexa Fluor 568 | Invitrogen | A-10042 | 1:500, IF |
| Donkey anti-mouse Alexa Fluor 568 | Invitrogen | A-10037 | 1:500, IF |
| Goat anti-rabbit Alexa Fluor 488 | Invitrogen | A-11008 | 1:500, IF |
| Donkey anti-mouse Alexa Fluor 647 | Invitrogen | A-3157 | 1:500, IF |
| Goat anti-rat Alexa Fluor 647 | Invitrogen | A-21247 | 1:500, IF; 1:50, flow cytometry |

| | | | |
|---|---|---|---|
| BrdU (anti-rat) | BioRad | MCA6144 | 1:1000, DNA fiber assay |
| IdU (anti-mouse) | Biosciences | 347580 | 1:1000, DNA fiber assay |
| Donkey anti-mouse Alexa Fluor 488 | Invitrogen | A-21202 | 1:500, DNA fiber assay |
| Goat anti-rat Alexa Fluor 568 | Invitrogen | A-11077 | 1:500, DNA fiber assay |
